# *Bacteroides*-derived isovaleric acid enhances mucosal immunity by facilitating intestinal IgA response in broilers

**DOI:** 10.1186/s40104-022-00807-y

**Published:** 2023-01-06

**Authors:** Xinkai Wang, Yifan Hu, Xiaoyan Zhu, Liyuan Cai, Muhammad Zahid Farooq, Xianghua Yan

**Affiliations:** 1 State Key Laboratory of Agricultural Microbiology, Hubei Hongshan Laboratory, Frontiers Science Center for Animal Breeding and Sustainable Production, College of Animal Sciences and Technology, Huazhong Agricultural University, Wuhan, 430070 Hubei China; 2 Department of Animal Sciences, University of Veterinary and Animal Sciences (Jhang Campus), Lahore, 54000 Pakistan

**Keywords:** *Bacteroides*, Chicken, IgA, Intestinal health, Isovaleric acid, Macrophage

## Abstract

**Background:**

The interaction between nutrition and immunity plays a vital role in nutrient digestion, absorption, and metabolism during poultry production. Recent studies showed that the gut microbiota contributes to the development of intestinal mucosal immunity. However, the mechanisms by which gut microbes regulate this process remain unclear.

**Methods:**

We compared the intestinal mucosal immunity and gut microbiota of Arbor Acre broilers (AA (lower mucosal immunity) and Chinese native Wuliang Mountain Black-bone chickens (WLMB) (higher mucosal immunity) using 16S rDNA sequencing, transcriptomic analysis, and immunoglobulin A (IgA) antibody repertoire sequencing. We then combined 16S rDNA sequencing with transcriptomics to identify the key microbes and found that they were positively correlated with IgA production. Next, we transplanted candidate microbes into 1-day-old broiler to explore their role in intestinal mucosal immunity. Finally, we verified the function of candidate microbial metabolites in regulating the immune function of macrophages and the intestinal-epithelial cells (IECs) using in vitro experiments.

**Results:**

WLMB performs stronger mucosal immunity than AA, including higher IgA levels, more diverse IgA antibody repertoire, and higher bacterial affinity. *Bacteroides* was identified as the key microbes related to the intestinal IgA response. *Bacteroides* transplantation could increase IgA concentration in the duodenal contents by enhancing the expression of IgA, polymeric immunoglobin receptor (*PIgR*), B cell-activating factor of the TNF family (*BAFF*), and activation-induced cytidine deaminase (*AID*) in the duodenum. Additionally, *Bacteroides*-derived isovaleric acid promoted M2 macrophage polarization of macrophage via mTOR/PPAR-γ/STAT3 signaling pathways and regulated the immunologic function of IECs to produce cytokines, including interleukin (IL)-10, IL-4, BAFF, and transforming growth factor-beta (TGF-β), thus promoting IgA production in B cells by facilitating *AID* expression.

**Conclusion:**

Our study revealed that *Bacteroides* modulate the intestinal IgA response and maintain gut health in broilers. *Bacteroides* may be a promising alternative as an immunomodulatory microbial agent for developing next-generation probiotics for broiler production.

**Supplementary Information:**

The online version contains supplementary material available at 10.1186/s40104-022-00807-y.

## Background

The intestinal mucosa is the first gateway for pathogen invasion and affects the ability of chickens to resist pathogen infections [1]. Immunoglobulin A (IgA)-related adaptive immunity plays a vital role in maintaining intestinal homeostasis [2]. IgA-producing B cells exist in the lamina propria and secrete bacteria-specific IgA, which is transported across the epithelium and secreted into the intestinal lumen [3]. IgA regulates bacterial colonization, assists antigen presentation, and neutralizes pathogens to shield hosts from enteric pathogens [2]. IgA is essential for maintaining the luminal compartmentalization of intestinal bacteria. However, the proportion of these bacteria depends on the specificity and diversity of IgA, which may capture bacteria in the mucus layer [4], promoting phagocytosis, and killing bacteria that invade the mucosa [5]. The lack of IgA leads to enhance the possibility of invasion by commensal bacteria [6]. A lack of activation-induced cytidine deaminase (AID) results in defects in class-switch recombination (CSR) and deficiency of IgA plasma cells, which leads to the aberrant expansion of gut microbiota [7].

Gut microbiota play an important role in maintaining intestinal homeostasis and promoting immune system maturation [8, 9]. IgA is the key node connecting gut microbiota with intestinal immunity, and its production depends on the complete complement of the gut microbiota and long-term stimulation [2]. Dendritic cells actively recruit small quantities of bacteria that penetrate the mucosal barrier or are present on the apical surfaces of epithelial cells [10]. Bacterial-laden dendritic cells transmit antigenic information to T cells and B cells, further inducing the differentiation of B cells into plasma cells, thereby producing bacterial-specific IgA [6]. In the intestinal mucosa, early B cell development is regulated by commensal microbiota, which influences gut immunoglobulin repertoires that continuously diversify gut IgA by changing the heavy chain (H), variable (V), diversity (D), and joining (J) gene segments that encode the VH domain of IgA [11]. Gut microbiota can act as antigens to stimulate IgA production, and their metabolites, especially short-chain fatty acids (SCFAs), have also been revealed to affect IgA CSR and IgA B cell differentiation into IgA-plasma cells [12]. In response to the gut microbiota, plasma cells continuously release IgA, which passes through intestinal epithelial cells (IECs), followed by intraluminal accumulation, and IgA specificity rapidly changes with microbial composition [13].

This study aimed to compare intestinal immunity between Arbor Acre broilers (AA) and Chinese native Wuliang Mountain Black-bone (WLMB) chickens. We further hypothesized that the microbiota of Chinese native chickens conveys an enhanced mucosal immune response. Thus, transplanting such microflora to modern broiler breeds can facilitate mucosal immunity and gut health. We combined 16S rDNA sequencing with transcriptomics to identify key microbes and found that they could regulate intestinal IgA responses.

## Materials and methods

### Animals

All experiments were approved by the Huazhong Agricultural University Animal Experimental Ethical Inspection of Laboratory Animal Centre (ID Number: HZAUCH-2019-014). All the methods were following with the approved guidelines. The samples were collected from male Arbor Acre broilers and Chinese native Wuliang Mountain Black-bone chickens at 60-day-old. AA were raised from commercial broiler farm and WLMB were raised from farm with a range of 0.05 km^2^, located in Kunming County, Yunnan Province, China.

A total of 72 1-day-age AA broilers with similar body weight were randomly divided into three groups including control group (CON), antibiotic vancomycin-, neomycin-, ampicillin-, and metronidazole group (VNAM), and intestinal microbiota transplantation group (IMT), with 6 replicate cages of 4 broilers per replicate cage. For CON group, 1 mL the phosphate buffer saline (PBS) was orally introduced into broilers from 1 d to 21 d. For VNAM group, 1 mg/mL neomycin, 1 mg/mL ampicillin, 0.5 mg/mL vancomycin, 1 mg/mL metronidazole were administered in the drinking water from 1 d to 21 d. For IMT group, duodenal contents were mixed with PBS, and then filtered through sterile gauze. Bacterial concentration was adjusted to 10^9^ CFU/mL, all operations were carried out under anaerobic conditions. 1 mL suspension was orally introduced into broilers from 1 d to 21 d. For sampling, 1 bird of average weight were selected from each replicate, after 12 h of overnight fasting.

A total of 72 1-day-age AA broilers with similar body weight were randomly divided into three groups including CON group, *Bacteroides caecicola* treatment group (BC) and *Bacteroides uniformis* treatment group (BU) with 6 replicate cages of 4 broilers per replicate cage. For CON group, 1 mL phosphate buffer saline (PBS) was orally introduced into broilers from 1 d to 21 d. *Bacteroides* culture solution was filtered and centrifuged to collect bacteria and resuspended them in PBS. Fanally,10^9^ CFU/mL BC or BU was orally introduced 1 mL into broilers from 1 d to 21 d, respectively. For sampling, 2 birds of average weight were selected from each replicate, after 12 h of overnight fasting.

For AA, the basal corn-soybean meal diets were formulated to meet the chickens nutrient requirements (China, NY/T 33–2004, 2004) [14] (Table S1). For WLMB, the diets were formulated to meet the WLMB nutrient requirements (China, DB5308/T 16.3–2014, 2014) [15] (Table S2). Feeding times for WLMB were at 8:00 h and 15:00 h, and had free access to water in grasslands and grove. For sampling, birds were slaughtered by cervical dislocation at 21 days of age. Intestinal contents were collected in sterile centrifuge tubes, and then frozen in liquid nitrogen immediately and stored at − 80 °C for intestinal microbiome analysis. The small intestine was divided into right size pieces and frozen in liquid nitrogen and stored at − 80 °C or frozen in opti-mum cutting temperature (O.C.T.) compound (Cat No. 4583, Sakura, Torrance, CA, USA).

### RT-qPCR

Animal tissues and cultured cells were quickly transferred into 1.5-mL centrifuge tubes containing Trizol reagent (Cat No.15596018, Invitrogen, CA, USA). Total RNA was extracted from the samples according to reagent instructions. The purity of RNA samples was detected with NanoDrop 2000 (Thermo, Massachusetts, USA). RNA integrity was detected by agarose gel electrophoresis. Total RNA (1 μg) was used to produce cDNA by Synthesis Kit (Cat No. RK20429, ABclonal, Wuhan, China). Genomic DNA was digested with the accompanying Dnase.

Primers were designed using BLAST [16] or Primer 3 [17] with GenBank sequences deposited on NCBI [18]. Primers were synthesized by Sangon Biotech Co., Ltd. (Shanghai, China), and the amplification efficiencies of all primers were between 95% and 105% (Table S3). Qbase + 3.3 (Biogazelle, Zwijnbeke, Belgium) software was used to optimize reference genes. β-actin and hydroxymethylbilane synthase (*HMBS*) were chosen as the reference genes in intestine, *GAPDH* and β-actin were chosen as the reference genes in HD-11 and IECs (geNorm M < 0.50, V_2/3_ < 0.15 threshold).

RT-qPCR was executed using SYBR Green Mix (Cat No. RK21206, ABclonal) on QuantStudio 6 PCR System (Applied Biosystems, Hammonton, NJ, USA). The thermocycler condition was as following, Step1, 95 °C for 10 min, Step2, 40 cycles of 95 °C for 5 s and 60 °C for 30 s, melting curves were collected from 60 °C to 90 °C. For *IL-2*, Step1, 95 °C for 3 min, Step2, 40 cycles of 95 °C for 5 s, 62 °C for 20 s, 72 °C for 30 s, melting curves were collected from 60 °C to 90 °C. The target gene expressions were normalized with the geometric mean of reference genes, relative mRNA expression levels were calculated using the 2^-ΔΔCT^ method [19].

### Reagents and antibodies

PE-conjugated anti-chicken IgA (Cat No.8330–09, SouthernBiotech, Birmingham, AL, USA), AF647-conjugated anti-chicken IgM (Cat No.8310–31, SouthernBiotech), NF-κB (Cat No.bs-0465R, Bioss, Beijing, China), PE-conjugated anti-chicken MHC II (Cat No.8345–09, SouthernBiotech), β-actin (Cat No.AC026, Abclonal), P70S6K1 (Cat No.A2190, Abclonal), phospho-P70S6K1 T389 (Cat No.AP1059, Abclonal), Goat anti-rabbit IgG (H + L) Alexa Fluor 594 (Cat No.A11012, Invitrogen), GW9662 (Cat No.HY-16578, MCE, Shanghai, China), S3I-201 (Cat No.SD4794, Beyotime Biotechnology, Shanghai, China).

### Immunofluorescence

Frozen sections (15 μm) and cultured cells were fixed with 4% paraformaldehyde for 15 min and washed twice with PBS. Triton X-100 (0.2%, v/v) was used to treat samples for 15 min on ice. Then the samples were incubated with primary antibody for 1 h and washed 3 times with PBS. Goat anti-rabbit IgG antibody (Cat No. A11037, Invitrogen) was used as the secondary antibody incubating for 1 h at 37 °C. The samples were stained with DAPI to detect cell nuclei. Images were acquired by confocal microscope (LSM 800, Carl Zeiss, Jena, Germany).

### ELISA

Intestinal contents or tissues samples were soaked in PBS (100 μL to 10 mg sample) and homogenized using homogenizer, then centrifuged at 12,000 × *g* for 10 min to remove small particles, and the supernatant was collected. Free IgA and IgM concentration were detected using chicken IgA, and IgM enzyme-linked immunosorbent assay (ELISA) quantitation Set (HUDING Biology, Shanghai, China).

### Fluorescent in situ hybridization

Frozen sections (15 μm) were fixed in 4% paraformaldehyde for 15 min, then heated at 60 °C for 20 min. Sections were dehydrated with 50%, 75%, 100% ethanol for 10 min, sequentially. Hybridization was performed overnight at 50 °C with 10 μg/mL universal bacterial probe (EUB388; 5′-[6-FAM]-GCTGCCTCCCGTAGGAGT-3′) in hybridization buffer (20% formamide, 20 mmol/L Tris–HCl (pH 7.4), 0.9 mol/L NaCl, 0.1% SDS). Then sections were washed 10 min in washing buffer 3 times (20 mmol/L Tris–HCl, pH 7.4, 0.9 mol/L NaCl) and 10 min in PBS. After blocking in block solution (PBS containing 5% BSA) for 30 min at 4 °C, sections were stained with antibody at 37 °C for 1 h. Finally, sections were washed 10 min in PBS 3 times, and images were acquired by confocal microscope (LSM880, Carl Zeiss).

### IgA mRNA sequencing and analyzing

Synthesize cDNA as described above. The chicken heavy-chain mRNA was searched in NCBI (Accession S40610). The consensus sequences of V region segment were used to design three pairs of primers (Table S4), and the primers were used to amplify the V(D) J region at the same time. PCR productions of 450 bp were purified using Gel Extraction. The samples were entrusted to Shanghai Biozeron Biological Technology Co. Ltd. for sequencing on the Illumina MiSeq PE250 platform. All tegs were analyzed by ImMunoGeneTics [20].

### Flow cytometry and fluorescence-activated cell sorting of IgA^+^ Bacteria

Frozen feces (100 mg) were placed in Lysing Matrix D tubes (MP Biomedicals, CA, USA) on ice and incubated in 1 mL PBS for 1 h, then homogenized for 5 s. The suspension was centrifuged (50 × *g* for 15 min, 4 °C) to remove large particles. 100 μL bacterial liquid was transferred into a new centrifuge tube. The samples were washed with 1 mL staining buffer (PBS, 1% (w/v) BSA) twice (8000 × *g* for 5 min, 4 °C), then were blocked in 100 μL staining buffer containing 20% normal mouse serum for 20 min on ice. Samples were stained in 100 μL staining buffer containing 10% PE-conjugated anti-chicken IgA for 30 min on ice, then were washed with 1 mL staining buffer 3 times and resuspended in 1 mL PBS. Samples were analyzed by CytoFlex (Beckman Coulter, CA, USA), and sorting of IgA-positive bacterial was performed using MoFlo XDP Sorter (Beckman Coulter), 2 million events of IgA-positive bacteria were collected. Unstained samples were used as a negative gate.

### Genomics DNA extraction

The total DNA of intestinal content was extracted by QIAamp PowerFecal DNA Kit (QIAGEN, Hilden, Germany). IgA-positive bacteria were mixed with 400 μL PBS, 250 μL 0.1 mm zirconia/silica beads (Biospec, Bartlesville, OK, USA), 300 μL lysis buffer (200 mmol/L NaCl, 200 mmol/L Tris, 20 mmol/L EDTA, pH 8), 200 μL 20% SDS and 500 μL DNA extraction reagent (25:24:1, pH 7.9, Solarbio). Then the mixture was homogenized twice for 2 min on ice, followed by centrifugation (6000 × *g*, 4 °C). Supernatant was transferred to a Phase Lock Gel Light (Cat No.WM5–2302820, TIANGEN, Beijing, China), one volume of DNA extraction reagent was added, and samples were mixed and centrifuged for 3 min (16,100 × *g*, room temperature). The aqueous phase was transferred into a new centrifuge tube and added with equal volume of isopropanol and 1/10 volume of NaOAc (3 mol/L, pH 5.5). The mixture was incubated at − 20 °C overnight and centrifuged for 20 min (16,100 × *g*, 4 °C). The precipitate was washed with 1 mL 100% ethanol and centrifuged for 3 min (16,100 × *g*, 4 °C), dried and resuspended in 100 mL TE buffer (50 °C, 30 min). The DNA was purified with TIANquick Mini Purification Kit (TIANGEN) [21].

### 16S rDNA sequencing and analyzing

30 ng template DNA was used to amplify V3–V4 regions of bacterial 16S rDNA by Primers 341F (5′-ACTCCTACGGGAGGCAGCAG-3′) and 806R (5′-GGACTACHVGGGTWTCTAAT-3′) with Illumina adapter, pad, and linker sequences (94 °C for 3 min, 30 cycles of 94 °C for 30 s, 56 °C for 45 s, 72 °C for 45 s and final extension for 10 min at 72 °C). The PCR products were purified. The libraries were used for sequencing on Illumina MiSeq platform following the standard pipelines of Illumina and generating 2 × 300 bp paired end reads (BGI, Shenzhen, China). From each sample, more than 38,000 × 2 raw reads were obtained. Filtering raw data to remove adaptors and low-quality bases, splice paired-end reads were to get the tags (FLASH, v1.2.11) [22]. We obtained more than 34,000 × 2 clean reads and got more than 34,000 tags in each sample. The tags were then clustered into operational taxonomic units (OUT) with a cutoff value of 97% threshold using UPARSE and chimera sequences were removed using UCHIME with the Gold database (v4.2.40) [23, 24]. The Ribosomal Database Project classifier (RDP) Classifier v.2.2 was used to assign taxonomy to representative OTUs with a minimum confidence threshold of 0.6 and OUTs were trained on the Greengenes database (v201305) by Quantitative Insights into Microbial Ecology (QIIME) (v1.8.0) [25]. Alpha and Beta diversity of OTUs were analyzed by MOTHUR (v1.31.2) and QIIME (v1.8.0) respectively [25, 26].

Phylogenetic beta diversity measures such as principal coordinates analysis (PCoA) was performed using the QIIME 2 [27]. Linear discriminant analysis (LDA) effect size (LEfSe) analysis was used to identify taxonomic biomarkers with LDA score > 2.0 and *P* < 0.05. Key biomarkers were defined with LDA score ≥ 4.0 and *P* < 0.05 [28]. Significant species and functions were determined by R (v3.4.1) based on Wilcox-test or Kruskal-test. Functions predicting of KEGG and COG were using the phylogenetic investigation of communities by reconstruction of unobserved states (PICRUSt) software.

### Transcriptomics analysis

Total RNA extraction, mRNA library construction and sequencing were entrusted to BGI China. The sequencing data were filtered by SOAPnuke (v1.5.2), including sequencing adapter. Low-quality reads whose base ratio (base quality less than or equal to 5) is more than 20% and reads whose unknown base (‘N’ base) ratio is more than 5% were removed [29]. HISAT2 (v2.0.4) was applied to map clean reads to the reference genome [30]. Clean reads were aligned to the reference coding gene set by Bowtie2 (v2.2.5) then RSEM (v1.2.12) was applied to calculate gene expression levels [31, 32]. The heatmap was drawn by pheatmap (v1.0.8). Differential expression analysis was performed using the DESeq2 (v1.4.5) with Q value ≤0.05 [33]. KEGG enrichment analysis of DEGs was performed by Phyper [34] based on Hypergeometric test. The significant levels of terms and pathways were corrected by Q value with Q value ≤ 0.05.

### Concentration determination of intestinal content SCFA

We determined the concentration of SCFAs in the intestinal content using gas chromatograph (GC) with the previous method [35]. Briefly, 500 mg of the intestinal content was transferred into a 1.5-mL centrifuge tube with 0.5 mL of methanol. After being vortexed for 30 s and centrifuged for 10 min (12,000 × *g*, 4 °C), 0.5 mL of supernatant was transferred to new centrifuge tube, added with 0.1 mL of 25% metaphosphoric acid and incubated overnight at 4 °C. After being centrifuged for 10 min (1200 × *g*, 4 °C), 0.5 mL of supernatant was analyzed using GC.

### Cell culture

HD-11 cells were cultured in DMEM/F12 medium with 5% FBS, 1% 100 U/mL penicillin/streptomycin (37 °C and 5% CO_2_). 19-day-old embryos of AA were used to isolate IEC. Small intestine was sliced into small pieces and incubated with DMEM containing 0.2 mg/mL collagenase IV at 37 °C for 40 min. Cells were collected by passaging supernatant through a 100 μm cell strainer. After washing with PBS, the crypts were cultured in DMEM/F12 with 5% FBS and 1% insulin-transferrin-selenium at 37 °C and 5% CO_2_. After reaching 80% confluence, medium was replaced with serum-free medium containing various combinations of isovaleric acid (IVA), GW9662 and S3I-201, which dissolved in DMSO. The control group was treated with DMSO.

### Western blotting

Proteins were separated in 8% SDS-polyacrylamide gels and transferred to NC membranes. The membranes were blocked in 5% BSA at room temperature for 1 h and incubated with primary antibody overnight at 4 °C. And then the membranes were incubated with secondary antibody for 1 h at room temperature and developed with enhanced chemiluminescence method (ECL). Chemiluminescence imager was used to obtain images.

### Statistical analysis

All experiments design and their replicates were described in each figure legend. All the data generated were examined for normal distribution. Significant analyses were performed using GraphPad Prism v.9 with the method of unpaired two-tailed Student’s *t* test. The data were shown as mean ± SEM. *P* < 0.05 was considered significant. * *P* < 0.05, ** *P* < 0.01, *** *P* < 0.001.

## Results

### WLMB presents a higher intestinal mucosal IgA response than AA

**Fig. 1:**
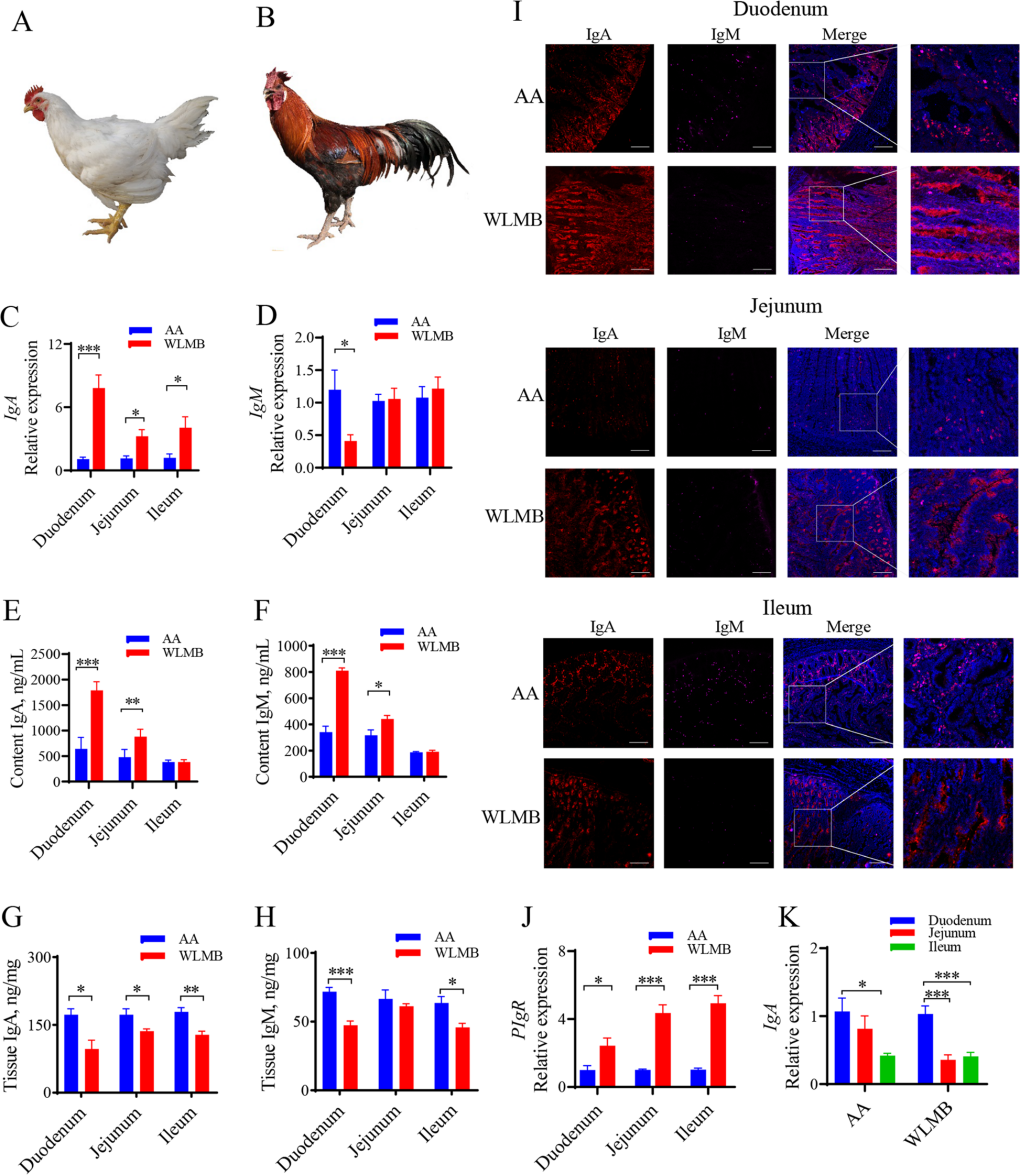
WLMB shows higher intestinal mucosal IgA response than AA. **A** and **B** AA and WLMB, respectively. **C** and **D** Gene expression levels of *IgA* and *IgM* in small intestine between AA and WLMB (*n* = 6). **E** to **H** Concentration of IgA and IgM in intestinal contents and intestinal tissues between AA and WLMB (*n* = 5). **I** Distribution of IgA (red) and IgM (purple) in intestine (blue) between AA and WLMB. Scale bars, 200 μm. **J** Gene expression level of *PIgR* in small intestine between AA and WLMB (*n* = 6). **K** Gene expression level of *IgA* in duodenum, Jejunum, and Ileum (*n* = 6). Data are means ± SEM; unpaired Student’s *t* test; β-actin and *HMBS* were used as the endogenous control for qPCR; * *P* < 0.05, ** *P* < 0.01, *** *P* < 0.001

To investigate the difference in intestinal mucosal immunity between commercial AA (Fig. 1A) and WLMB (Fig. 1B), we detected mucosal immune-related immunoglobulin (Ig) in the small intestine of 60-day-old chickens. At the gene expression level, WLMB had a higher expression level of *IgA* than AA (*P* < 0.05) (Fig. 1C), whereas AA had a higher immunoglobulin M (*IgM*) expression level in the duodenum than WLMB (*P* < 0.05) (Fig. 1D). Then, ELISA was used to detect IgA and IgM at the protein level. WLMB had higher IgA and IgM concentrations in the intestinal contents (*P* < 0.05) but lower concentrations in the intestinal tissue (*P* < 0.05) than AA (Fig. 1E to H).

Confocal images showed obvious IgA staining outside the intestinal villi of WLMB and a larger number of dots of IgA and IgM staining in intestinal villi of AA (Fig. 1I). Therefore, we detected the expression level of intestinal polymeric Ig receptor (*PIgR*) was higher in WLMB than in AA (*P* < 0.05) (Fig. 1J), revealing that the increased expression level of *PIgR* endowed WLMB with an enhanced immunoglobulin secretion ability. Additionally, we compared the relative expression level of *IgA* in different intestinal segments and observed that the expression level of *IgA* was higher in the duodenum than in other intestinal segments (*P* < 0.05) (Fig. 1K), indicating that WLMB has a higher mucosal IgA response in the duodenum than AA.

### Intestinal IgA of WLMB has a higher bacterial affinity than that of AA

**Fig. 2:**
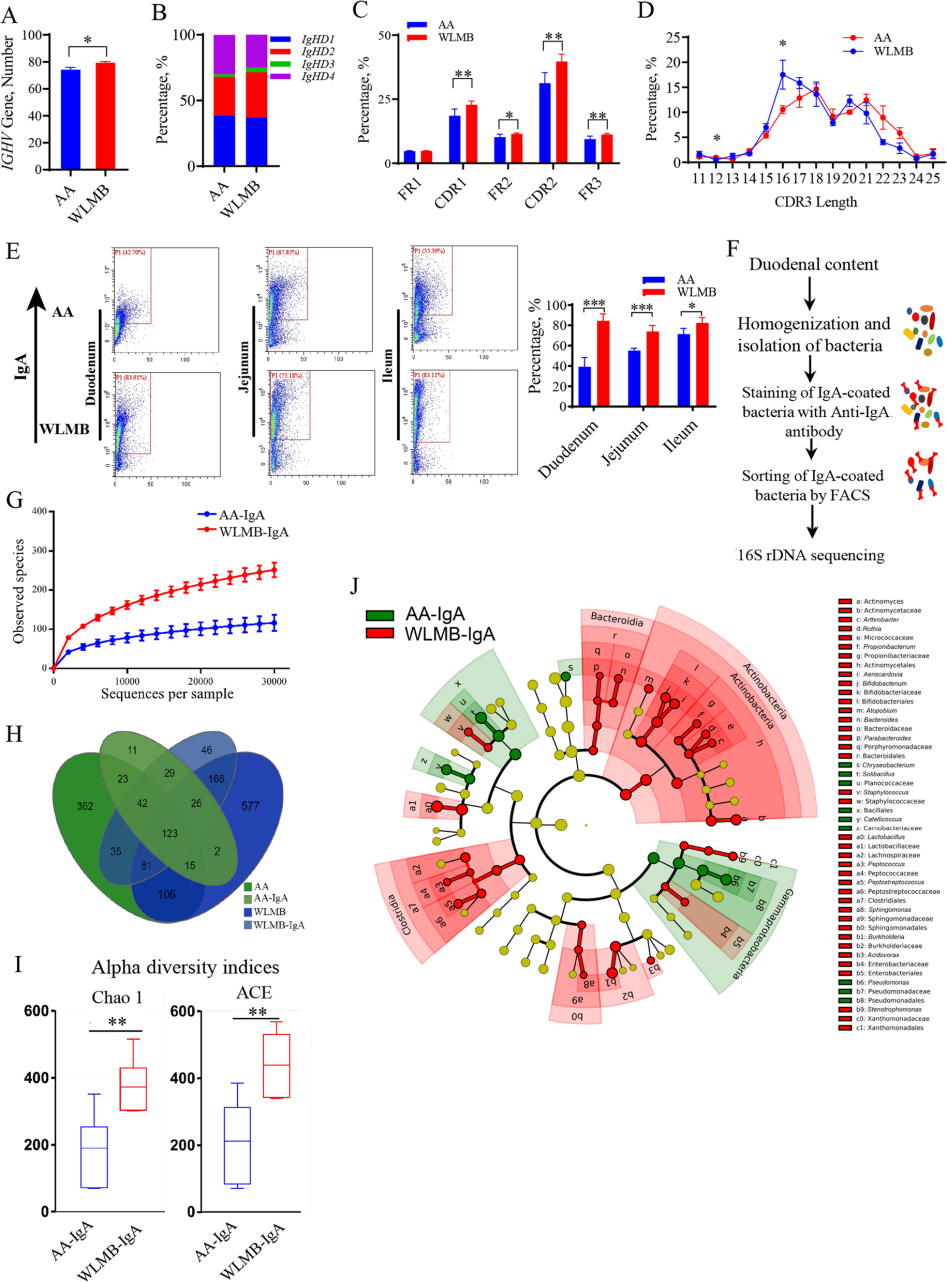
Intestinal IgA of WLMB has a higher bacterial affinity than that of AA. **A** Number of *IGHV* gene used in duodenal IgA between AA and WLMB. **B** Usage frequency of *IGHD* gene in duodenal IgA. **C** Frequency of mutations targeting the FR1, CDR1, FR2, CDR2, and FR3 regions of duodenal IgA. **D** The distribution of different sized CDR3 fragments for IGH amino acid sequences in duodenal IgA. **E** Flow cytometric analysis of the proportion of IgA-coated bacteria in small intestine, the IgA^+^ gates were determined follows negative control. **F** Overview of 16S rDNA sequencing of IgA-coated bacteria in the duodenum based on FACS. **G** Rarefaction curves of observed OTUs. **H** Venn diagram depicting the number of OTUs identified in total bacterial and IgA-coated bacterial in duodenum. **I** Alpha diversity indices of IgA-AA and IgA-WLMB. **J** Cladogram based on LEfSe. Biomarker taxons were heighted by colored circles, circle’s diameter is relative to the abundance of biomarkers. Data are means ± SEM (*n* = 6); unpaired Student’s *t* test; * *P* < 0.05, ** *P* < 0.01, *** *P* < 0.001

To determine the differences in duodenal IgA between AA and WLMB, we performed high-throughput sequencing of the duodenal IgA antibody repertoire. WLMB possessed a more diverse immunoglobulin heavy chain variable region (*IGHV*) gene used in IgA mRNA than AA (*P* < 0.05) (Fig. 2A). However, no significant differences were detected in the usage frequency of each *IGHV* gene (Additional file 5). Significant differences between AA and WLMB were observed in the usage frequency of the immunoglobulin heavy constant delta (*IGHD*) 2 and *IGHD*3 genes (*P* < 0.05) (Fig. 2B). Moreover, WLMB had a higher frequency of mutations targeting the complementary determining region (CDR) 1, framework region (FR) 2, CDR2, and FR3 in duodenal IgA than AA (*P* < 0.05) (Fig. 2C). Additionally, we found that the CDR3 length in WLMB tended to be shorter than that in AA (Fig. 2D).

Flow cytometric analysis showed that the proportion of IgA-coated gut microbiota in the intestine of WLMB was significantly higher than that of AA (*P* < 0.05) (Fig. 2E). 16S rDNA sequencing was used to analyze the composition of duodenal bacteria and IgA-coated bacteria in the duodenum based on fluorescence-activated cell sorting (FACS) (Fig. 2F). Rarefaction curves showed the availability of sufficient reads to cover all OTUs present in the samples (Fig. 2G). There were 271 OTUs in the IgA-coated bacteria of AA (AA-IgA) and 548 in the IgA-coated bacteria of WLMB (WLMB-IgA), with 787 in AA and 857 in WLMB (Fig. 2H). The alpha diversity Chao 1 and ACE indices showed that the individual microbiota diversity was higher in WLMB-IgA than in AA-IgA (*P* < 0.05) (Fig. 2I). With IgA-coated bacteria sorting, 7 and 10 families were enriched in the guts of AA and WLMB, respectively. However, there was no statistical difference in the relative abundance of these families between the AA and WLMB groups (Additional file 6). Furthermore, the relative abundances of 10 families (> 0.05%) in WLMB-IgA were higher than that in AA-IgA. However, the relative abundance of one family in AA-IgA was higher than that in WLMB-IgA (Additional file 6). The results suggested that IgA in the intestine of WLMB can specifically bind to more families than that in the intestine of AA and could bind to most of the families enriched from the AA gut. We further predicted that IgA could specifically select bacteria and enrich low-abundance species. The LEfSe analysis showed that Clostridia, Bacteroidia, and Actinobacteria were marker phyla in the microbiota of WLMB-IgA (Fig. 2J). Actinomycetaceae, *Actinomyces*, Bifidobacteriaceae, *Bifidobacterium*, Lactobacillaceae, *Lactobacillus*, Burkholderiaceae, *Burkholderia*, and *Sphingomonas* were identified as the key biomarkers in WLMB-IgA at the genus and family levels, whereas Planococcaceae, *Solibacillus*, Carnobacteriaceae, *Catellicoccus*, Pseudomonadaceae, and *Pseudomonas* were identified as the key biomarkers in AA-IgA (Fig. 2J, Additional file 6). These data demonstrate that WLMB possesses IgA with a high affinity for enhancing bacterial coating, which may depend on the highly diverse CDR3 region.

### IgA-production-related pathways are upregulated in the duodenum of WLMB

**Fig. 3:**
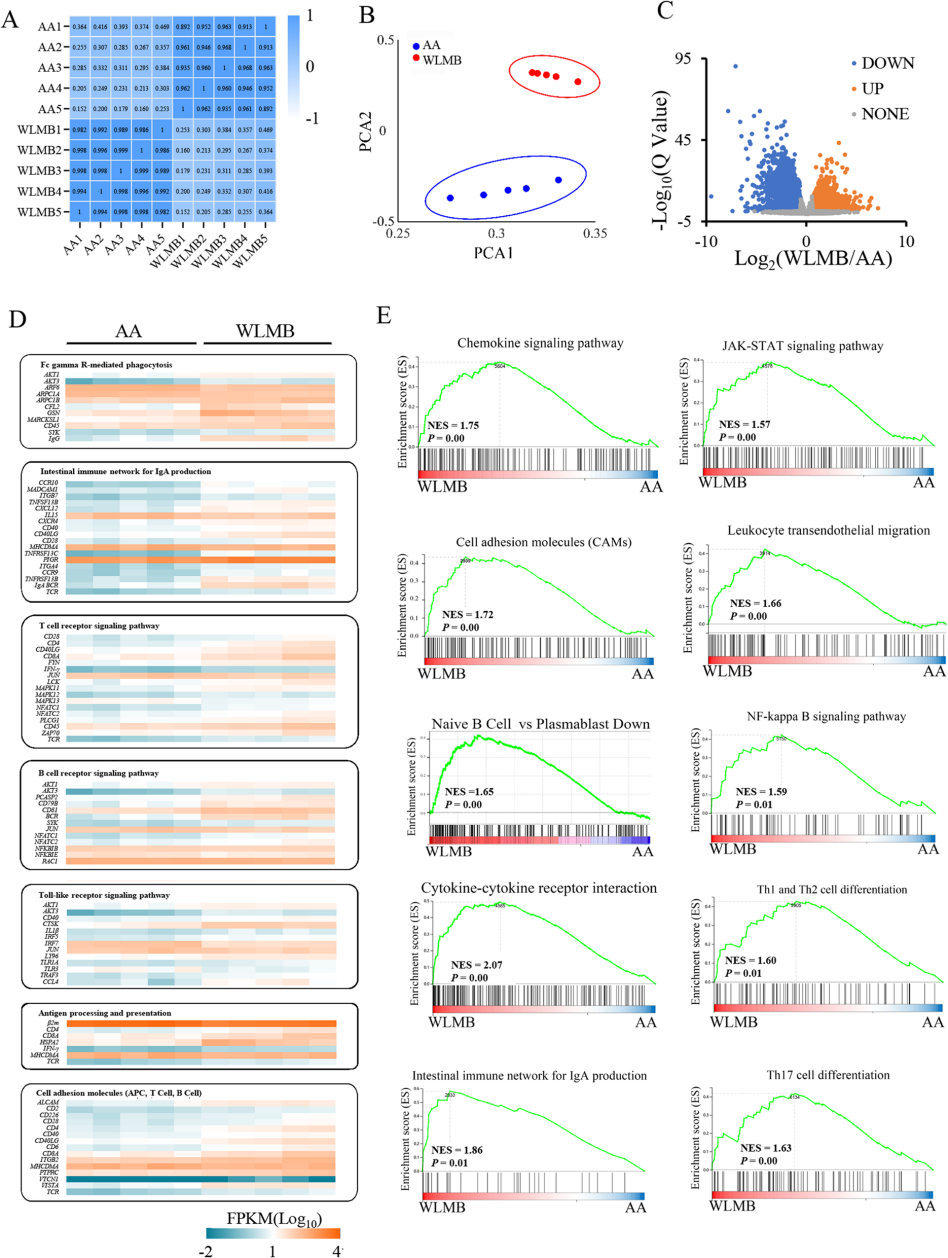
IgA-production-related pathways are upregulated in the duodenum of WLMB. **A** Sample correlation heatmap based on the Pearson’s correlation coefficients of all gene expression between every two samples. **B** PCA plot of gene expression between AA and WLMB. **C** Volcano plot of genes, Q value < 0.05 and |log_2_(fold change)| > 0.5850 as the threshold parameters. **D** Heatmap of DEGs in KEGG pathways related to IgA production, based on fragments per kilobase per million (FPKM). **E** Enrichment plots of DEGs from the gene set enrichment analysis (GSEA). NES, normalized enrichment score

Transcriptome analysis was performed to explore the high production of IgA in the duodenum of WLMB. Pearson’s correlation coefficients and principal component analysis (PCA) showed a high similarity of intraclass samples and differences between AA and WLMB (Fig. 3A and B). There were 6600 differentially expressed genes (DEGs) between AA and WLMB, including 3476 upregulated genes and 3124 downregulated genes in WLMB compared with AA (Fig. 3C). Additionally, 176 KEGG pathways were significantly enriched (Q value < 0.05). DEGs in IgA production-related pathways, including Fc gamma R-mediated phagocytosis, intestinal immune network for IgA production, T cell receptor signaling pathway, B cell receptor signaling pathway, Toll-like receptor signaling pathway, antigen processing and presentation, and cell adhesion molecules (CAMs), were significantly higher in WLMB than those in AA, as shown by the IgA production-related signaling molecules (CD40LG, BAFF, IL1B, TGFB1 and IFNG), signaling receptors (TLR1, CD40, CD4, TCR, BCR and BAFFR), and IgA secretion-related protein PIgR (Fig. 3D). Gene set enrichment analysis (GSEA) revealed significant enrichment in the chemokine signaling pathway, Jak-STAT signaling pathway, cell adhesion molecules (CAMs), leukocyte transendothelial migration, naive B cell vs plasmablast down, NF-kappa B signaling pathway, cytokine-cytokine receptor interaction, Th1 and Th2 cell differentiation, intestinal immune network for the IgA production pathway, and Th17 cell differentiation gene sets (Fig. 3E). These results demonstrated significant differences in intestinal IgA production-related pathways between AA and WLMB.

### Gut Microbiota regulate the duodenal IgA response

**Fig. 4:**
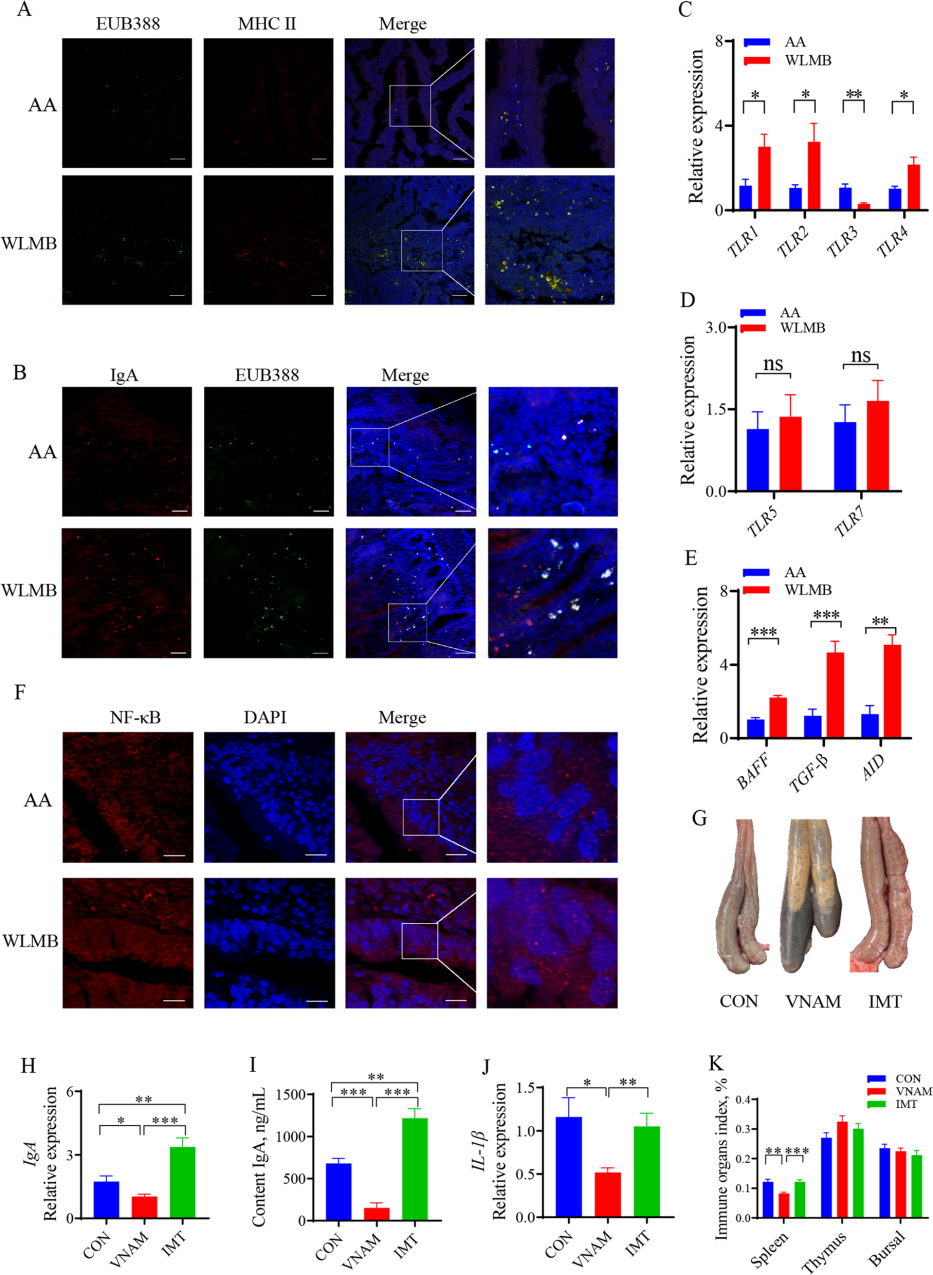
Gut Microbiota regulate the duodenal IgA response. **A** Confocal images showed the tissues (blue), MHC II (red) and FISH probes EUB388-FAM targeted total bacteria (green) in duodenum sections, Scale bars, 100 μm. **B** Confocal images showed the tissues (blue), IgA (red) and FISH probes EUB388-FAM targeted total bacteria (green) in duodenum sections, Scale bars, 50 μm. **C–E** Gene expression levels in duodenum of AA and WLMB (*n* = 6). **F** Confocal images showed NF-κB (red) and nucleus (blue) in duodenum, Scale bars, 10 μm. **G** Morphology of the cecum and cecal contents. **H** Gene expression level of IgA in duodenum (*n* = 6). **I** Concentration of IgA in duodenal contents (*n* = 6). **J** Gene expression level of *IL-1β* in duodenum (*n* = 6). **K** Immune organ index among control, VNAM, and IMT (*n* = 6). Data are means ± SEM; unpaired Student’s *t* test; β-actin and *HMBS* were used as the endogenous control for qPCR. * *P* < 0.05, ** *P* < 0.01, *** *P* < 0.001

Fluorescence in situ hybridization (FISH) and immunostaining showed high level of co-localization of bacteria and antigen resenting cells (APCs) in WLMB intestinal villi but low level of co-localization in AA (Fig. 4A), which demonstrated that the APCs of WLMB had a powerful ability to capture gut bacteria. Partial luminal antigen sampling was dependent on IgA (Fig. 4B). RT-qPCR showed that the expression levels of Toll-like receptor (*TLR*) 1, *TLR2*, and *TLR4* in WLMB was significantly higher than those in AA (*P* < 0.05), whereas the expression level of *TLR3* in WLMB was significantly lower than that in AA (*P* < 0.05) (Fig. 4C), no significant differences were observed in expression levels of *TLR5* and *TLR7* (*P* > 0.05) (Fig. 4D). The expression of transforming growth factor-β (*TGF-β*) and B cell–activating factor of the TNF family (*BAFF*) were higher in WLMB than in AA (*P* < 0.05) (Fig. 4E). These cytokines activate *AID* expression (Fig. 4E), which plays an essential role in CSR and somatic hypermutation (SHM) of immunoglobulin genes in B cells. Confocal microscopy images showed the migration of nuclear factor kappa B (NF-κB) into the nucleus to promote the expression level of *PIgR* in intestinal epithelial cells (Fig. 4F).

To determine whether the gut microbiota of WLMB is a key factor in enhancing the IgA response, intestinal microbiota transplantation (IMT) was carried out from the duodenum of WLMB into 1-day-old broilers, and VNAM treatment was used to establish a gut model lacking microbe. VNAM treatment significantly altered the morphology of the cecum and cecal contents, indicating that the model was successful (Fig. 4G). IMT significantly enhanced the expression of *IgA* (*P* < 0.05) and increased IgA concentration in the duodenal contents (*P* < 0.05). The lack of microbiota reduced gut IgA response (Fig. 4H and I). However, no significant differences were detected for expression level of the interleukin (*IL*)-1β and thymus, bursal, and thymus indices (Fig. 4J and K), suggesting that the gut microbiota of WLMB can induce a high-level IgA response in the small intestine.

### Microbial diversity and community composition in the duodenum of AA and WLMB

**Fig. 5:**
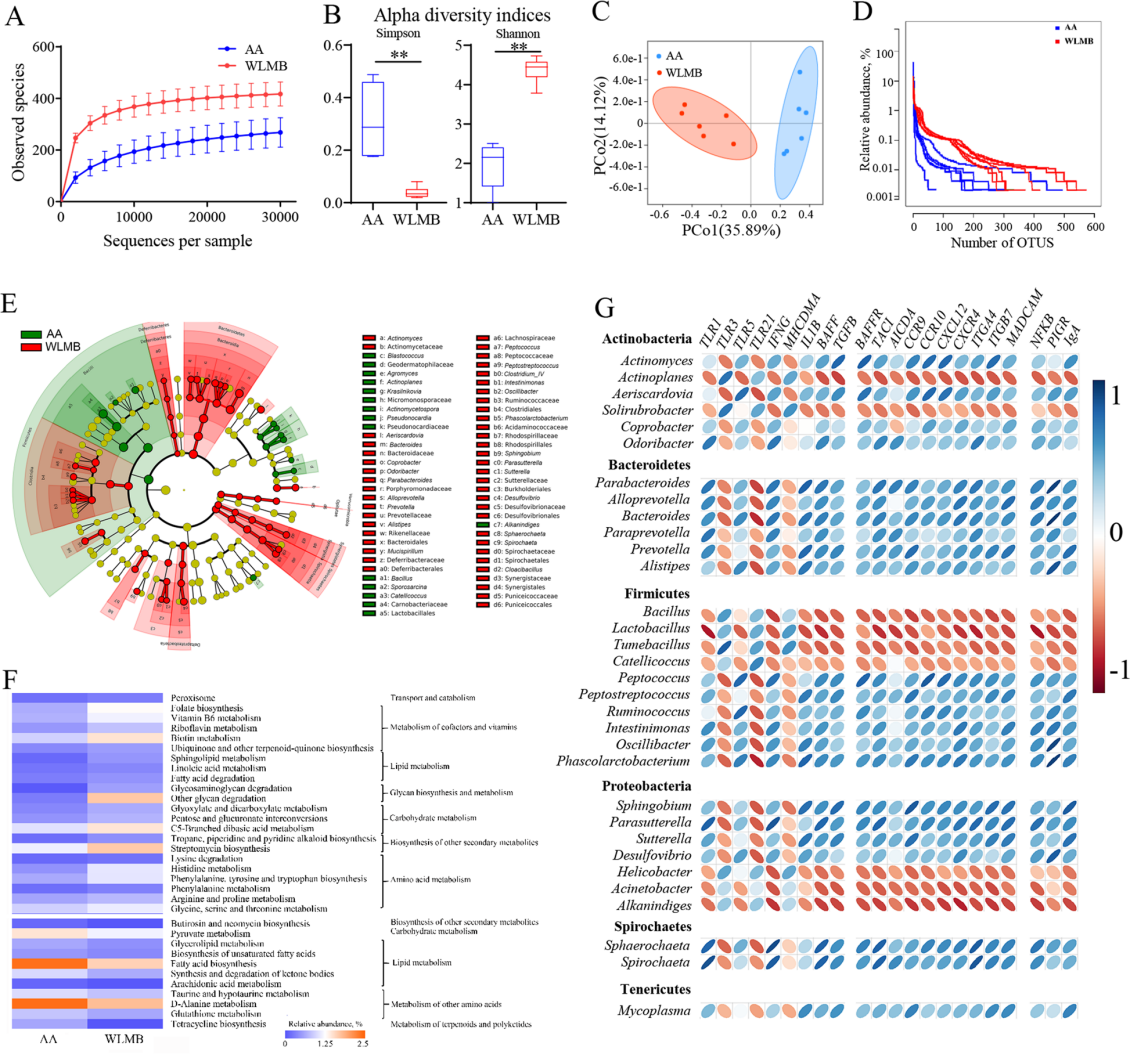
Microbial community composition and functional analysis in duodenum between AA and WLMB. **A** Rarefaction curves of observed OTUs. **B** Alpha diversity indices between AA and WLMB (*n* = 6). **C** Principal coordinates analysis (PCoA) plot of duodenal microbiota between AA and WLMB (unweighted UniFrac distance matrix). **D** Rank curves of each sample. **E** LEfSe analysis was performed to identify microbiota taxa that were significantly difference between AA and WLMB. Biomarker taxa were heighted by colored circles, circle’s diameter is relative to the abundance of biomarkers. **F** Duodenum bacterial function profiles. Heatmap showed the differential abundant of KEGG pathways. **G** Heatmap of the correlation coefficient between the genera of microbiota and the IgA-related DGEs. Data are means ± SEM; unpaired Student’s *t* test; ** *P* < 0.01

Rarefaction curves indicated the availability of sufficient reads to cover all species in the samples (Fig. 5A). Alpha diversity analysis showed that the gut microbiota in WLMB exhibited an improved Simpson and Shannon indices compared with that in AA (*P* < 0.05) (Fig. 5B). The beta diversity presented by the principal coordinates analysis (PCoA) plot showed that the gut microbiota in WLMB was obviously different from that in AA (Fig. 5C), and AA had a higher proportion of dominant flora and lower diversity than WLMB (Fig. 5D).

We determined the difference in bacterial composition between AA and WLMB at different taxonomic levels (Additional file 7). At the phylum level, five phyla in AA and three phyla in WLMB were identified, with a relative abundance of > 1%. WLMB possessed a higher relative abundance of Bacteroidetes and Spirochaetes and a lower relative abundance of Firmicutes than AA. At the genus level, ten abundant genera were identified in AA and eight abundant genera were identified in WLMB. The relative abundances of *Bacteroides* (17.93%), *Prevotella* (2.54%), *Actinomyces* (2.45%), *Sphaerochaeta* (2.25%), *Aeriscardovia* (1.80%), *Desulfovibrio* (1.62%), and *Parabacteroides* (1.48%) were significantly higher in WLMB than in AA, whereas the relative abundances of *Lactobacillus* (43.43%), *Catellicoccus* (24.33%), *Helicobacter* (14.53%), *Acinetobacter* (1.039%), and *Bacillus* (1.10%) were significantly higher in AA than in WLMB. LEfSe analysis showed that the relative abundance of 75 bacterial clades at all taxonomic levels were significantly different between AA and WLMB (Fig. 5E). Three key biomarkers at the genus level, *Bacteroides*, *Sphaerochaeta*, and *Prevotella*, were identified in the WLMB (Additional file 7).

Compared with AA, WLMB exhibited an increased relative abundances of functions in the metabolism of cofactors and vitamins, including folate biosynthesis, vitamin B_6_ metabolism, riboflavin metabolism, biotin metabolism, ubiquinone, and other terpenoid-quinone biosynthesis, which are important for maintaining normal immune physiological functions in animals. Compared with WLMB, AA exhibited increased functions in amino acid metabolism, such as lysine degradation, Histidine metabolism, phenylalanine, tyrosine and tryptophan biosynthesis, phenylalanine metabolism, arginine and proline metabolism, glycine, serine, and threonine metabolism, which contribute to the growth efficiency of animals (Fig. 5F).

Thereafter, we explored the correlation between gut microbiota and genes in IgA production-related pathways. The results showed that Bacteroidetes and Spirochaetes were positively correlated with IgA production (Fig. 5G), therefore, we predicted that *Bacteroides* is the key microbe in the duodenum of WLMB that mainly induces intestinal IgA response.

### Intestinal immunologic changes in response to colonization with *Bacteroides caecicola* and *Bacteroides uniformis*

**Fig. 6:**
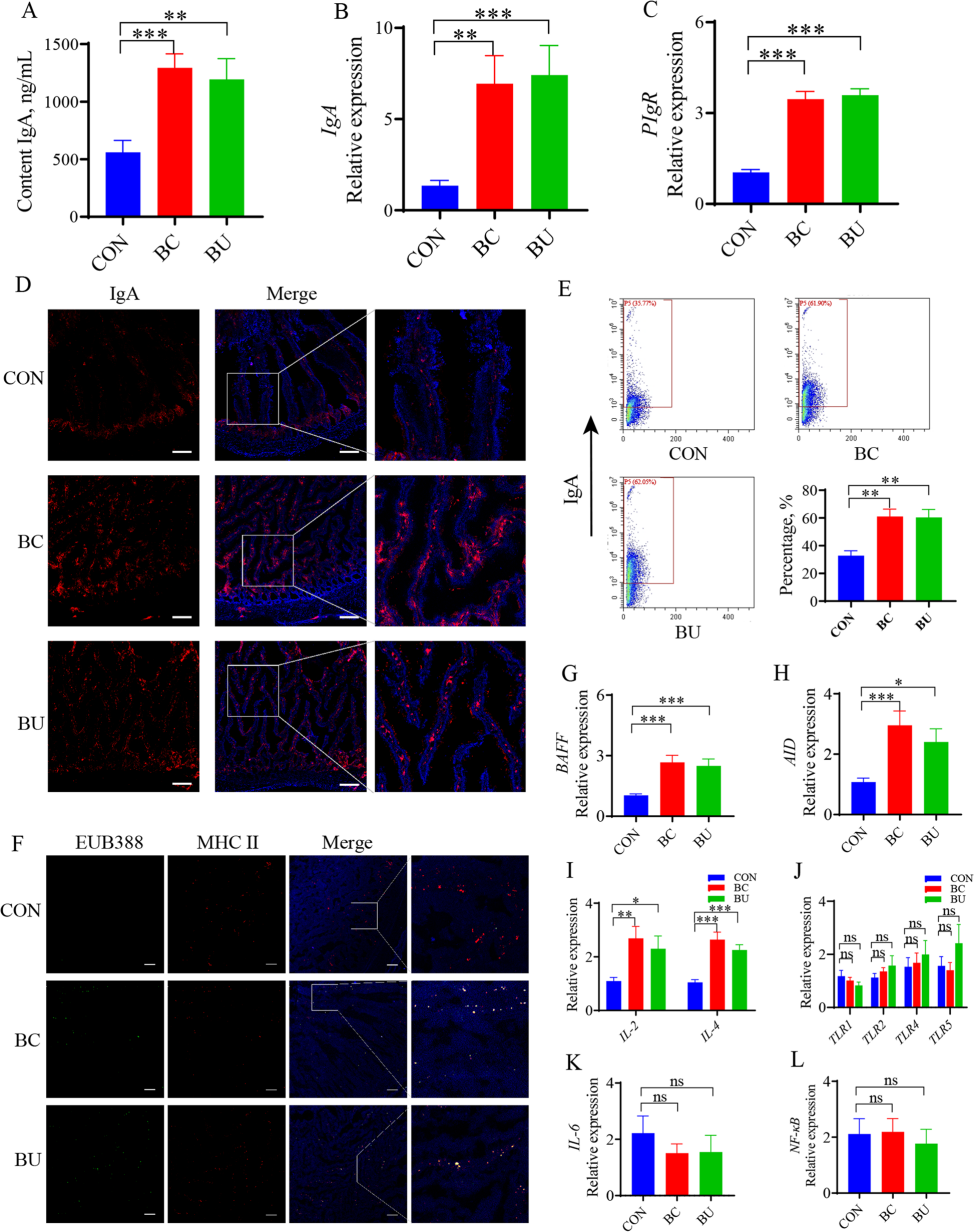
Intestinal immune performance in response to colonization with *Bacteroides caecicola* and *Bacteroides uniformis*. **A** Concentration of IgA in duodenal contents (*n* = 12). **B** and **C** Gene expression levels of *IgA* and *PIgR* in duodenum (*n* = 12). **D** Distribution of IgA (red) in duodenal tissues (blue). Scale bars, 200 μm. **E** Flow cytometric analysis the proportion of IgA-coated bacteria in duodenal contents, the IgA^+^ gates were determined follows negative control (*n* = 6). **F** Confocal images showed the tissues (blue), MHC II (red) and FISH probes EUB388-FAM targeted total bacteria (green) in duodenum, scale bars, 50 μm. **G–L** Gene expression levels of in duodenum (*n* = 12). Data are means ± SEM; unpaired Student’s *t* test; β-actin and *HMBS* were used as the endogenous control for qPCR; * *P* < 0.05, ** *P* < 0.01, *** *P* < 0.001

To evaluate the effects of *Bacteroides* on the development of intestinal mucosal immune system, *Bacteroides caecicola* and *Bacteroides uniformis* were isolated from the duodenum of WLMB and orally introduced into 1-day-old broilers from 1 to 21 days of age. The results of ELISA and RT-qPCR showed that *Bacteroides* improved the concentration of IgA in the duodenal contents by enhancing the expression of *IgA* and *PIgR* (*P* < 0.05) (Fig. 6A to C). We observed an increase in the number of IgA-positive cells in the intestinal villi (Fig. 6D). Flow cytometric analysis revealed that *Bacteroides* significantly increased the proportion of IgA-coated bacteria in the duodenal contents (*P* < 0.05) (Fig. 6E), indicating that *Bacteroides* improved the bacterial affinity of IgA. Confocal images showed that *Bacteroides* increased the colocalization of bacteria and APCs in the intestinal villi (Fig. 6F). Both *Bacteroides* strains elevated the expression levels of *BAFF* and *AID* in the duodenum (*P* < 0.05), while increased the expression levels of *IL-2* and *IL-4* (*P* < 0.05) (Fig. 6G to I). Furthermore, no significant difference was detected in the gene expression levels of *TLRs*, *IL-6*, and *NF-κB* after treatment with *Bacteroides* (*P* > 0.05) (Fig. 6J to L). These results suggest that BC and BU are potential probiotics that may stimulate the production of IgA through their metabolites.

### Regulation of immunologic function for macrophages by *Bacteroides* metabolites

**Fig. 7:**
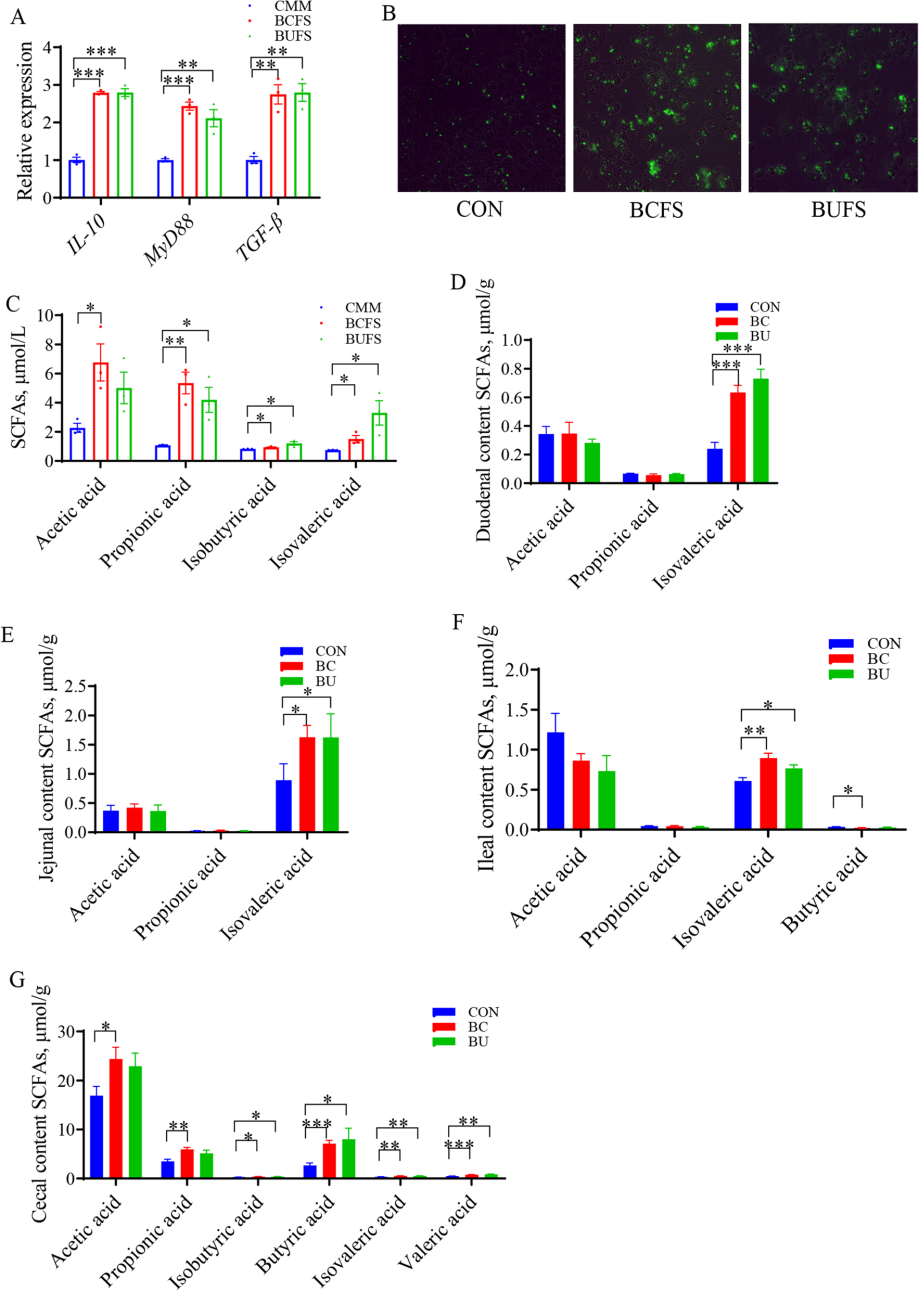
*Bacteroides* metabolites regulate the immunologic function of macrophages. **A** Gene expression levels of *IL-10*, *MyD88* and *TGF-β* in HD-11 treated for 5 h (*n* = 3). **B** HD-11were treated for 5 h, then were cultured in DMEM containing GFP-labeled *Escherichia coli* for 2 h. Phagocytosis was detected by fluorescence microscopy (20 ×). **C** Concentration of acetic acid, propionic acid, isobutyric acid, and isovalerate acid in CMM, BCFS, and BUFS (*n* = 3). **D–G** Concentration of SCFAs in intestinal contents (*n* = 6). Data are means ± SEM, β-actin and *GAPDH* were used as the endogenous control for qPCR; * *P* < 0.05, ** *P* < 0.01, *** *P* < 0.001

BC and BU were cultured in chopped meat medium (DSMZ Medium 78, CMM) for 48 h. *Bacteroides caecicola* fermentation supernatant (BCFS) and *Bacteroides uniformis* fermentation supernatant (BUFS) were used to treat the chicken macrophage cell line (HD-11). After 5 h of treatment, cytokine analysis revealed that the expression levels of *IL-10*, myeloid differentiation primary response 88 (*MyD88*), and *TGF-β* on HD-11 cells were notably elevated (*P* < 0.05) (Fig. 7A). Furthermore, these treatments improved the phagocytic ability of HD-11 cells (Fig. 7B), therefore, we predicted that the metabolites of *Bacteroides* might have a positive immunomodulatory effect. We detected SCFAs in *Bacteroides* fermentation supernatant using GC, and the results showed that *Bacteroides* produce acetic acid, propionic acid, isobutyric acid, and isovaleric acid (IVA) (*P* < 0.05) in the chopped meat medium (Fig. 7C).

Additionally, we detected SCFA concentrations in the intestinal contents of broilers treated with BC and BU. BC and BU significantly increased the concentration of isovaleric acid in the small intestine (*P* < 0.05) (Fig. 7D to F). However, they also increased the concentrations of acetic acid, propionic acid, isobutyric acid, butyric acid, isovaleric acid, and valeric acid in the cecum (*P* < 0.05) (Fig. 7G). Thus, the *Bacteroides* metabolite isovaleric acid mainly regulates the immune function in the small intestine.

### *Bacteroides*-derived isovaleric acid regulates macrophage polarization and immunologic function of IECs

**Fig. 8:**
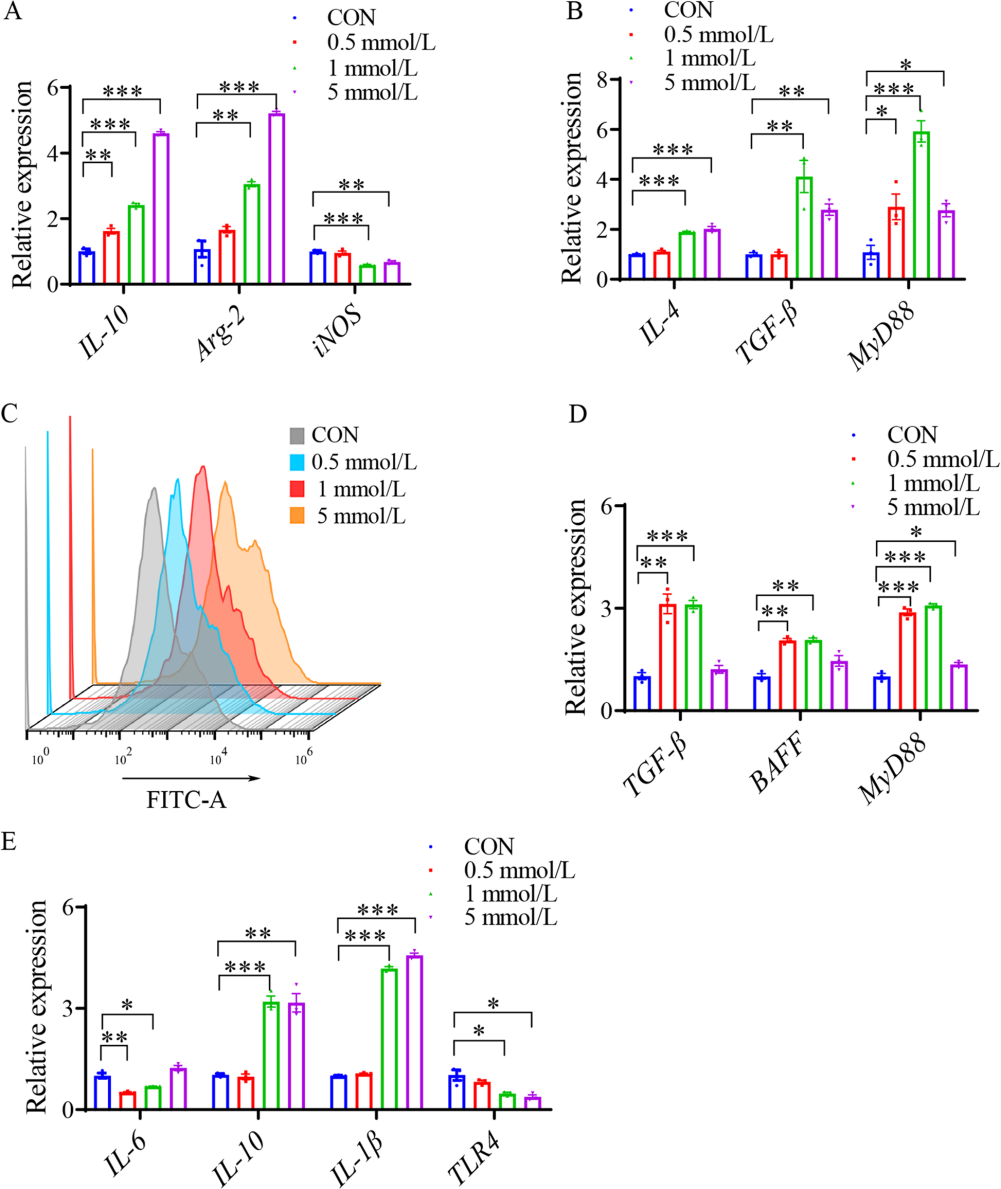
*Bacteroides*-derived isovaleric acid regulates macrophage polarization and immunologic function of IECs. **A** and **B** Gene expression levels of *IL-10*, *Arg-2*, *iNOS*, *IL-4*, *TGF-β* and *MyD88* in HD-11 treated with different doses of isovalerate acid for 5 h (*n* = 3). **C** HD-11were treated for 5 h with different doses of isovalerate acid, then were cultured in DMEM containing GFP-labeled *Escherichia coli* for 2 h. Phagocytosis was detected by flow cytometry (*n* = 3). **D** and **E** Gene expression levels of *TGF-β*, *BAFF*, *MyD88*, *IL-6*, *IL-10*, *IL-1β*, and *TLR4* in IEC treated with different doses of isovalerate acid for 5 h (*n* = 3). Data are means ± SEM; unpaired Student’s *t* test; β-actin and *GAPDH* were used as the endogenous control for qPCR. * *P* < 0.05, ** *P* < 0.01, *** *P* < 0.001

To study whether IVA regulates the function of macrophages, we treated the HD-11 cell line with different doses of IVA (0.5, 1, and 5 mmol/L) for 5 h. RT-qPCR results showed that the expression levels of *IL-10*, arginases (Arg)-2, and *TGF-β* increased (*P* < 0.05), but the expression level of inducible nitric oxide synthase (*iNOS*) decreased (*P* < 0.05) after treatment with IVA*,* which conformed to the key features of classically activated macrophages (M1) to alternatively activated macrophages (M2) (Fig. 8A and B). Polarization of M2 macrophages elevated the expression levels of *IL-4*, *TGF-β*, and *MyD88* (*P* < 0.05) (Fig. 8B) and enhanced phagocytic activity (Fig. 8C), thereby promoting the proliferation and differentiation of B cells and production of IgA. IVA promoted the expression of *TGF-β*, *BAFF*, and *MyD88* in IECs (*P* < 0.05) (Fig. 8D). In addition, IVA treatment exerted an immunomodulatory function and inhibited intestinal inflammation by increasing the gene expression levels of *IL-10* and *IL-1β* (*P* < 0.05) and reducing the gene expression levels of *IL-6* and *TLR4* (*P* < 0.05) (Fig. 8E). Overall, these data suggest that *Bacteroides*-derived IVA improves mucosal immunity and maintains intestinal homeostasis by regulating M2 polarization of macrophages and improving the expression of specific cytokines in macrophages and IECs.

### Isovaleric acid regulates macrophage M2 polarization via the mTOR/PPAR-γ/STAT3 signaling pathway

**Fig. 9:**
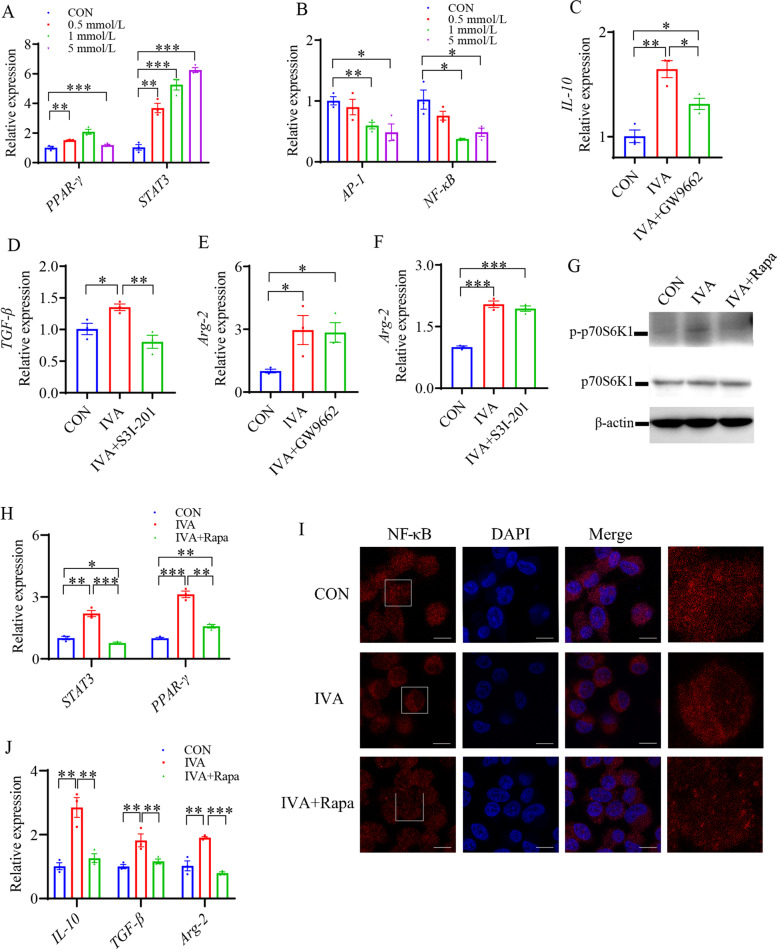
Isovaleric acid regulates the polarization of macrophages via the mTOR signaling pathway. **A **and **B** Gene expression levels of *PPAR-γ*, *STAT3*, *AP-1*, and *NF-κB* in HD-11 treated with different dose of IVA for 5 h (*n* = 3). **C** Gene expression level of *IL-10* in HD-11 treated with 1 mmol/L IVA or 1 mmol/L IVA + 10 μmol/L GW9662 for 5 h (*n* = 3). **D** Gene expression level of *TGF-β* in HD-11 after treated with 1 mmol/L IVA or 1 mmol/L IVA + 50 μmol/L S3I-201 for 5 h (*n* = 3). **E** Gene expression level of *Arg-2* in HD-11 treated with 1 mmol/L IVA or 1 mmol/L IVA + 10 μmol/L GW9662 for 5 h (*n* = 3). **F** Gene expression level of *Arg-2* in HD-11 after treated with 1 mmol/L IVA or 1 mmol/L IVA + 50 μmol/L S3I-201 for 5 h (*n* = 3). **G** Western blot analyses for p-p70 S6K1 T389 and total p70 S6K1 in HD-11 cells treated with 1 mmol/L IVA or 1 mmol/L IVA + 250 nmol/L rapamycin for 5 h. **H** Gene expression levels of *STAT3* and *PPAR-γ* in HD-11 treated with 1 mmol/L IVA or 1 mmol/L IVA + 250 nmol/L rapamycin for 5 h (*n* = 3). **I** Confocal images showed NF-κB (red) and nucleus (blue) in HD-11 treated with 1 mmol/L IVA or 1 mmol/L IVA + 250 nmol/L rapamycin for 5 h (*n* = 3), scale bars, 10 μm. **J** Gene expression levels of *IL-10*, *TGF-β*, and *Arg-2* in HD-11 treated with 1 mmol/L IVA or 1 mmol/L IVA + 250 nmol/L rapamycin for 5 h (*n* = 3). Data are means ± SEM; unpaired Student’s *t* test; β-actin and *GAPDH* were used as the endogenous control for qPCR. * *P* < 0.05, ** *P* < 0.01, *** *P* < 0.001

To elucidate the mechanism by which IVA regulates macrophage polarization and cytokine secretion, HD-11 cells were treated with various doses of IVA. After 5 h of treatment, we detected the gene expression levels of macrophage polarization-related transcription factors using RT-qPCR. The results showed that IVA elevated the expression levels of M2 polarization-related transcription factors peroxisome proliferator-activated receptor gamma (*PPAR-γ*) and signal transducer and activator of transcription (STAT) 3 (*P* < 0.05) (Fig. 9A) and inhibited the expression levels of M1 polarization-related transcription factors activating protein-1 (*AP-1*) and *NF-κB* (*P* < 0.05) (Fig. 9B). Afterward, we used 1 mmol/L IVA as a physiological concentration in *Bacteroides*-treated duodenum and investigated the effects of PPAR-γ and STAT3 on the expression levels of M2 macrophage marker genes. HD-11 cells were treated with GW9662 and S3I-201 inhibitors of PPARγ and STAT3, respectively. GW9662 significantly reduced the IVA-induced high expression of *IL-10* (*P* < 0.05), and S3I-201 significantly reduced the IVA-induced high expression of *TGF-β* (*P* < 0.05) (Fig. 9C and D). However, GW9662 and S3I-201 did not affect the expression level of *Arg-2* (*P* < 0.05) (Fig. 9E and F).

Recent studies have shown that IVA can activate the mTOR pathway, which is required for the M2 polarization of macrophages in humans and mice [36, 37]. Therefore, we speculate that IVA regulates the M2 polarization of macrophages by activating the mTOR pathway in poultry. We treated HD-11 cells with IVA and rapamycin and detected the activity of mTOR and the gene expression levels of the M2 polarization-related transcription factors *PPARγ* and *STAT3*. Western blotting results showed that IVA significantly increased the phosphorylation level of 70-kDa ribosomal protein S6 kinase (p70S6K), whereas treatment with rapamycin decreased the IVA-induced phosphorylation level of p70S6K (Fig. 9G). RT-qPCR results showed that rapamycin inhibited IVA-induced high expression of *PPAR-γ* and *STAT3* (*P* < 0.05) (Fig. 9H). IVA inhibited NF-κB activity, whereas rapamycin decreased the inhibitory effect (Fig. 9I). The results indicated that IVA regulated the expression levels of *PPAR-γ* and *STAT3* and the activity of NF-κB via the mTOR signaling pathway. To further examine the regulation of macrophage polarization by IVA through mTOR signaling, we measured the mRNA expression levels of *IL-10*, *TGF-β*, and *Arg-2*, the results showed that rapamycin inhibited the IVA-induced significant high expression of *IL-10*, *TGF-β*, and *Arg-2* (*P* < 0.05) (Fig. 9J). These results suggest that IVA activates the transcription factors STAT3 and PPAR-γ by activating mTOR signaling pathway to regulate the polarization of M2 macrophages and promote the secretion of IgA production-related cytokines.

## Discussion

In this study, WLMB exhibited higher mucosal immunity than commercial AA, as shown by the higher expression and secretion of IgA with higher bacterial affinity. Previous study has shown that genetic background can affect immune performance in poultry [38]. In addition to genetic background, age, nutrients, and gut microbes can affect the gut immune performance of poultry [39, 40]. Our study focused on the effects of gut microbiota on intestinal immune performance. Gut microbiota has been widely proven to be highly correlated with gut immune performance [41]. As a key microbe in WLMB, *Bacteroides* plays a vital role in intestinal IgA response. Independent of the bacterial TLR ligands, which induce IgA responses through the binding of TLRs, *Bacteroides*-derived IVA induces M2 polarization of macrophages and the expression of *IL-10*, *IL-4*, *TGF-β*, and *BAFF* to promote IgA response by activating the mTOR/PPAR-γ/STAT3 signaling pathway in the small intestine.

Transcriptomic analysis showed that, compared with AA, the T cell-independent IgA mucosal immune response, including TGF-β and BAFF activation of BAFF-R and transmembrane activator and calcium-modulator and cyclophilin-ligand interactor (TACI), was upregulated in the WLMB duodenum to mediate the expression of *AID* in B cells. AID-dependent IgM-to-IgA CSR and IgA SHM promote the production of IgA B cells and the differentiation of IgA B cells into IgA-plasma cells [42, 43]. A lack of AID causes the absence of hypermutated IgA and a decrease in the bacteria-binding capacity of IgA, further leading to an imbalance in the intestinal flora [7, 42]. The antigen-binding specificity of IgA depends on the hypervariable CDR3 region, which could be modified by antigen stimuli, including bacterial ligands [44]. The size of CDR3 affects the structure of an antigen-binding site, thereby affecting its antigen affinity [44]. Lower CDR3 regions in WLMB tend to be more hydrophilic and may be advantageous in the initiation of immune responses [45]. The *IGHD* gene encodes most CDR3, and its rearrangement leads to differences in CDR3. This process may depend on the high expression of *AID* [46]. The difference in the diversity index of duodenal IgA-coated bacteria between AA and WLMB also reflected the difference in IgA affinity.

Intestinal immune performance is regulated by diverse intestinal commensal bacteria [47]. Therefore, the screening of key microbiota in native chickens may improve commercial broiler production. We found that WLMB possessed a higher relative abundance of Bacteroidetes and Spirochaetes and a lower relative abundance of Firmicutes. Rosshart et al. [48] and Wilmore et al. [49] also reported that wild mice with high intestinal and serum immunity possess a higher relative abundance of Bacteroidetes and Proteobacteria than SPF mice. *Bacteroides*, an abundant genus in the gut, has recently been recommended as a next-generation probiotic candidate [50]. In vivo experiments showed that BC and BU significantly induced the expression of *AID* and promoted the production of IgA in the duodenum. The absence of an intestinal TLR pathway activated by BC and BU through microbe-associated molecular patterns indicates that *Bacteroides* may drive IgA response via metabolites and directly stimulate it as an antigen. Feeding a soluble fiber-rich diet increases the abundance of *Bacteroides* in the gut and elevates the gene expression level of *AID* in B cells [51]. Furthermore, by fermenting fiber, *Bacteroides* can produce several types of SCFAs that are taken up by IECs and diffuse into the lamina propria, where their effects are induced [52, 53]. Acetate induces the production of retinoic acid by dendritic cells via G protein–coupled receptor (GPR) 43 to promote B cell CSR and IgA production in mice [12]. Propionate can increase the population of Foxp3^+^ IL-10-producing cTregs and the proliferative capacity of cTregs [54]. Butyrate can promote the production of RegIIIc and β-defensins in IECs via the mTOR/STAT3 signaling pathway [55]. After treating broilers with *Bacteroides*, we found that IVA was the only SCFA that was upregulated in the small intestine.

Our study revealed the function of IVA in promoting the immunocompetence and M2 polarization of macrophages. M1 macrophages are considered proinflammatory [56], whereas M2 macrophages are considered anti-inflammatory and are related to tissue remodeling, immunomodulatory functions, and efficient phagocytic activity [56]. M2 polarization of macrophages depends on the activation of the transcription factors STAT3, PPAR-γ, and STAT6 [56, 57]. In this study, we found that IVA induced the expression of *STAT3* and *PPAR-γ* and inhibited the expression of *NF-κB* and *AP-1*. Furthermore, IVA significantly activates the mTOR signaling pathway, a core regulator of immune cell metabolism and function required for M2 polarization of macrophages [36]. The inhibition of the mTOR signaling pathway inhibited the expression of M2 macrophage markers *IL-10*, *TGF-β*, and *Arg-2*. IL-10 and TGF-β are regulated by PPARγ and STAT3, respectively. Through IVA stimulation, the high expression of *IL-4*, *IL-10*, and *TGF-β* is critical for T cell-independent IgA CSR, and it promotes the proliferation, differentiation, and IgA production of B cells [58, 59]. However, increased phagocytic activity with M2 polarization could promote antigen uptake and presentation. Activated macrophages express high levels of the antigen-presenting molecule major histocompatibility complex (MHC).

## Conclusion

The present study demonstrated that the gut microbiota of WLMB could promote the development of intestinal mucosal immunity, including higher IgA levels, more diverse IgA antibody repertoires, and higher bacterial affinity, in broilers. *Bacteroides* was identified as the key gut microbe in WLMB which has a critically important function in modulating the intestinal IgA response and maintaining intestinal health. Their metabolite isovaleric acid regulates the immunologic function of macrophages and IECs to produce cytokines via mTOR/PPAR-γ/STAT3 signaling pathway, including IL-10, IL-4, BAFF, and TGF-β, thus promoting IgA production in B cells by facilitating *AID* expression. As an immunomodulatory microbial agent, *Bacteroides* may be a promising alternative for developing next-generation probiotics in broiler production.

## Supplementary Information

**Additional file 1: Table S1.** Composition and nutrient levels of the experimental diets for AA broilers (as-fed basis)

**Additional file 2: Table S2.** Composition and nutrient levels of the experimental diets for WLMB (as-fed basis)

**Additional file 3: Table S3.** RT-qPCR primers in this study

**Additional file 4: Table S4.** IgA sequencing primers in this study

**Additional file 5.** Supplementary data of IgA mRNA sequencing

**Additional file 6.** Supplementary data of 16S rDNA sequencing in IgA + Bacteria

**Additional file 7.** Supplementary data of microbial community composition in the duodenum between AA and WLMB

## Data Availability

All Sequence data have been deposited in the NCBI Sequence Read Archive (SRA) under accession numbers PRJNA770257 (Transcriptome) (https://www.ncbi.nlm.nih.gov/sra/PRJNA770257) and PRJNA770462 (16S rDNA Sequencing) (https://www.ncbi.nlm.nih.gov/sra/PRJNA770462). All data generated or analyzed during this study can be made available by the corresponding author upon reasonable request.

## Abbreviations

AA: Arbor Acre broilers
AID: Activation-induced cytidine deaminase
AP-1: Activating protein-1
APCs: Antigen presenting cells
BAFF: B cell-activating factor of the TNF family
BC: *Bacteroides caecicola*
BCFS: *Bacteroides caecicola* fermentation supernatant
BU: *Bacteroides uniformis*
BUFS: *Bacteroides uniformis* fermentation supernatant
CDR: Complementary determining region
CON: Control group
CSR: Class-switch recombination
DEGs: Differentially expressed genes
ELISA: Enzyme-linked immunosorbent assay
FACS: Fluorescence-activated cell sorting
FISH: Fluorescence in situ hybridization
GC: Gas chromatograph
HD-11: Macrophage cell line
IECs: Intestinal epithelial cells
Ig: Immunoglobulin
IgA: Immunoglobulin A
IGHD: Immunoglobulin heavy constant delta
IGHV: Immunoglobulin heavy chain variable region
IgM: Immunoglobulin M
IL: Interleukin
IMT: Intestinal microbiota transplantation
iNOS: Inducible nitric oxide synthase
IVA: Isovaleric acid
LDA: Linear discriminant analysis
LEfSe: Linear discriminant analysis effect size
M1: Classically activated macrophages
M2: Alternatively activated macrophages
mTOR: Mechanistic target of rapamycin
MyD88: Myeloid differentiation primary response 88
NF-κB: Nuclear factor kappa B
OUT: Operational taxonomic units
p70S6K: 70-kDa ribosomal protein S6 kinase
PBS: Phosphate buffer saline
PCA: Principal component analysis
PCoA: Principal coordinates analysis
PICRUSt: Phylogenetic investigation of communities by reconstruction of unobserved states
PIgR: Polymeric Ig receptor
PPAR-γ: Peroxisome proliferator-activated receptor gamma
SCFAs: Short-chain fatty acids
STAT: Signal transducer and activator of transcription
TACI: Transmembrane activator and calcium-modulator and cyclophilin-ligand interactor
TGF-β: Transforming growth factor-β
TLR: Toll-like receptor
VNAM: Vancomycin-, neomycin-, ampicillin-, and metronidazole group
WLMB: Wuliang Mountain Black-bone chickens
