# Supplementary material for: Bacteroides-derived isovaleric acid enhances mucosal immunity by facilitating intestinal IgA response in broilers
Source: J Anim Sci Biotechnol. 2023 Jan 6;14:4. doi: 10.1186/s40104-022-00807-y (PMC9817248; doi:10.1186/s40104-022-00807-y)
Supplement: Supplementary file 1 — Additional file 1: Table S1. Composition and nutrient levels of the experimental diets for AA broilers (as-fed basis) [file 40104_2022_807_MOESM1_ESM.docx]

**Table S1** Composition and nutrient levels of the experimental diets for AA broilers (as-fed basis)

|  | 1–21days | 22–42days | 43–60days |
| --- | --- | --- | --- |
| **Ingredients, %** |  |  |  |
| Ground corn | 48.46 | 54.37 | 60.56 |
| Soybean oil | 5.00 | 5.50 | 5.50 |
| Soybean meal | 41.89 | 35.70 | 29.70 |
| Sodium chloride | 0.30 | 0.30 | 0.30 |
| Limestone | 1.22 | 1.00 | 0.81 |
| Calcium phosphate | 1.85 | 1.85 | 1.85 |
| 98.5% *L*-Lysine | 0.10 | 0.10 | 0.10 |
| 98% *DL*-Methionine | 0.17 | 0.17 | 0.17 |
| Premix^1^ | 1.01 | 1.01 | 1.01 |
| **Calculated composition, %** |  |  |  |
| Metabolizable energy, MJ/kg | 12.71 | 13.03 | 13.26 |
| Crude protein | 23.94 | 21.45 | 19.07 |
| Lysine | 1.47 | 1.30 | 1.13 |
| Methionine | 0.53 | 0.50 | 0.47 |
| Threonine | 0.92 | 0.82 | 0.73 |
| Tryptophan | 0.31 | 0.27 | 0.24 |
| Methionine + cystine | 0.92 | 0.85 | 0.79 |
| Calcium | 1.137 | 1.04 | 0.95 |
| Total phosphorous | 0.82 | 0.80 | 0.78 |
| Available phosphorous | 0.55 | 0.53 | 0.52 |
| **Analysed composition^2^, %** |  |  |  |
| Crude protein | 23.03 | 20.97 | 18.37 |
| Calcium | 1.12 | 0.93 | 0.96 |
| Ash | 2.37 | 2.41 | 2.23 |
| Phosphorous | 0.78 | 0.75 | 0.73 |

^1^Premix contained per kg: vitamin A, 10,000 IU; vitamin D_3_, 2500 IU; vitamin E, 18.75 mg; vitamin K_3_, 0.5 mg; vitamin B_1_, 2.5 mg, vitamin B_2_, 6.25 mg; vitamin B_6_, 2.5 mg; vitamin B_12_, 18.75 μg; nicotinic acid, 25.00 mg; pantothenic calcium, 12.50 mg; folic acid, 1.25 mg; biotin, 100 μg; choline chloride, 800 mg; Fe, 78 mg (as iron sulfate monohydrate); Mn, 80 mg (as manganous oxide); Zn, 60 mg (as zinc oxide); Cu, 8 mg (as copper sulfate pentahydrate); I, 0.4 mg (as calcium iodate); and Se, 0.2 mg (as sodium selenite). Phytase (per kilogram of diet): 1000 FTU

^2^Analysed by near-infrared spectroscopy (Lengguang technology, S450)
