# Supplementary material for: Bacteroides-derived isovaleric acid enhances mucosal immunity by facilitating intestinal IgA response in broilers
Source: J Anim Sci Biotechnol. 2023 Jan 6;14:4. doi: 10.1186/s40104-022-00807-y (PMC9817248; doi:10.1186/s40104-022-00807-y)
Supplement: Supplementary file 2 — Additional file 2: Table S2. Composition and nutrient levels of the experimental diets for WLMB (as-fed basis) [file 40104_2022_807_MOESM2_ESM.docx]

**Table S2** Composition and nutrient levels of the experimental diets for WLMB (as-fed basis)

|  | 1–28days | 29–60days |
| --- | --- | --- |
| **Ingredients, %** |  |  |
| Ground corn | 30.0 | 35.00 |
| Wheat bran | 15.00 | 10.00 |
| Rice bran | 20.00 | 26.00 |
| Soybean meal(exp.) | 26.00 | 22.30 |
| Soybean oil | 5.40 | 3.00 |
| Limestone | 1.00 | 1.00 |
| Calcium phosphate | 1.50 | 1.50 |
| 98% *DL*-Methionine | 0.10 | 0.20 |
| Premix^1^ | 1.00 | 1.00 |
| **Calculated composition, %** |  |  |
| Metabolizable energy, MJ/kg | 11.77 | 11.60 |
| Crude protein | 18.52 | 17.46 |
| Lysine | 0.97 | 0.91 |
| Methionine | 0.39 | 0.48 |
| Methionine + cystine | 0.70 | 0.77 |
| Calcium | 0.92 | 0.90 |
| Total phosphorous | 1.16 | 1.26 |
| Available phosphorous | 0.51 | 0.51 |
| **Analysed composition^2^, %** |  |  |
| Crude protein | 18.63 | 17.54 |
| Calcium | 1.01 | 0.93 |
| Phosphorous | 0.48 | 0.42 |

^1^Premix contained per kg: vitamin A, 10,000 IU; vitamin D_3_, 2500 IU; vitamin E, 18.75 mg; vitamin K_3_, 0.5 mg; vitamin B_1_, 2.5 mg, vitamin B_2_, 6.25 mg; vitamin B_6_, 2.5 mg; vitamin B_12_, 18.75 μg; nicotinic acid, 25.00 mg; pantothenic calcium, 12.50 mg; folic acid, 1.25 mg; biotin, 100 μg; choline chloride, 800 mg; Fe, 78 mg (as iron sulfate monohydrate); Mn, 80 mg (as manganous oxide); Zn, 60 mg (as zinc oxide); Cu, 8 mg (as copper sulfate pentahydrate); I, 0.4 mg (as calcium iodate); and Se, 0.2 mg (as sodium selenite). Phytase (per kilogram of diet): 1000 FTU

^2^Analysed by near-infrared spectroscopy (Lengguang technology, S450)
