# Supplementary material for: Bacteroides-derived isovaleric acid enhances mucosal immunity by facilitating intestinal IgA response in broilers
Source: J Anim Sci Biotechnol. 2023 Jan 6;14:4. doi: 10.1186/s40104-022-00807-y (PMC9817248; doi:10.1186/s40104-022-00807-y)
Supplement: Supplementary file 3 — Additional file 3: Table S3. RT-qPCR primers in this study [file 40104_2022_807_MOESM3_ESM.docx]

**Table S3** RT-qPCR primers in this study

| Gene | Product size, bp | Forward primer | [Reverse](javascript:;) primer | Reference | Amplification efficiency, % |
| --- | --- | --- | --- | --- | --- |
| *HMBS* | 131 | GGCTGGGAGAATCGCATAGG | TCCTGCAGGGCAGATACCAT | [1] | 98.30 (60 ℃) 97.60 (62℃) |
| β-actin | 101 | CACAGATCATGTTTGAGACCTT | CATCACAATACCAGTGGTACG | [2] | 98.11 (60 ℃) 99.16 (62℃) |
| *IgM* | 98 | GCATCAGCGTCACCGAAAGC | TCCGCACTCCATCCTCTTGC | [3] | 97.25 |
| *IgA* | 192 | GTCACCGTCACCTGGACTACA | ACCGATGGTCTCCTTCACATC | [3] | 98.51 |
| *PIgR* | 173 | TCATCGACACGTATGGGAAAA | CAGCTCCTTCGTTTCTCCATA | NM_001044644 | 95.80 |
| *TLR4* | 131 | TTCAGAACGGACTCTTGAGTGG | CAACCGAATAGTGGTGACGTTG | [NM_001030693.1](https://www.ncbi.nlm.nih.gov/nuccore/NM_001030693.1) | 103.57 |
| *TLR3* | 148 | ACTGCGGAATCTGACTGTCC | TCCTCCTGGGTTTGCACATT | [XM_025149682.2](https://www.ncbi.nlm.nih.gov/nuccore/XM_025149682.2) | 103.72 |
| *TLR5* | 192 | TTGTGTTGTGACCAGGCAGT | GGCCAACGCAGATATCGACT | [NM_001024586.1](https://www.ncbi.nlm.nih.gov/nuccore/NM_001024586.1) | 102.25 |
| *TLR1* | 238 | GCTGTGTCAGCATGAGAGGA | GTGGTACCTCGCAGGGATAA | [NM_001007488.4](https://www.ncbi.nlm.nih.gov/nuccore/NM_001007488.4) | 97.26 |
| *TLR2* | 246 | GAAAGTTCCCCCTTTTCCAG | AGAGTGCAGAAGGTCCCTGA | [NM_001161650.2](https://www.ncbi.nlm.nih.gov/nuccore/NM_001161650.2) | 102.09 |
| *TLR7* | 231 | GAGATCGGTGACGCTGAGTT | CCCAAGATCCAACACCGTCA | [NM_001011688.2](https://www.ncbi.nlm.nih.gov/nuccore/NM_001011688.2) | 102.73 |
| *IL-4* | 82 | GTGCCCACGCTGTGCTTAC | AGGAAACCTCTCCCTGGATGTC | [3] | 99.46 |
| *IL-2* | 219 | AGTCTTACGGGTCTAAATCACAC | GGACAGCAGATTAGTTAGCCA | [4] | 97.33 (62℃) |
| *IL-6* | 106 | AAATCCCTCCTCGCCAATCT | CCCTCACGGTCTTCTCCATAAA | [NM_204628.1](https://www.ncbi.nlm.nih.gov/nuccore/NM_204628.1) | 98.31 |
| *IL-1β* | 104 | CATCACCAACCAACCCGA | ACGAGATGGAAACCAGCAA | [NM_204524.1](https://www.ncbi.nlm.nih.gov/nuccore/NM_204524.1) | 97.95 |
| *IL-10* | 88 | CGCTGTCACCGCTTCTTCA | TCCCGTTCTCATCCATCTTCTC | [5] | 100.77 |
| *NF-κB* | 100 | GCACAACGCCTCTTCACATA | GGCTCAAAGTTCTCAACGTG | XM_015285415 | 97.09 |
| *AP-1* | 188 | TTTAGTCGCCAGGGGAAACT | GGCACAGCAGAAATAGAGGC | [NM_001006261.1](https://www.ncbi.nlm.nih.gov/nuccore/NM_001006261.1) | 98.03 |
| *PPAR-γ* | 189 | AGGCGATCTTGACAGGAAA | TAATCTCCTGCACTGCCTCC | [NM_001001460.1](https://www.ncbi.nlm.nih.gov/nuccore/NM_001001460.1) | 98.84 |
| *STAT3* | 156 | ACAACTACAGACTCGGCAGC | CTCTCCACCACGAAAGCACT | [NM_001030931.2](https://www.ncbi.nlm.nih.gov/nuccore/NM_001030931.2) | 96.06 |
| *iNOS* | 241 | TGGGTGGAAGCCGAAATA | GTACCAGCCGTTGAAAGGAC | [3] | 96.13 |
| *TGF-β* | 86 | ACCTCGACACCGACTACTGCTT | ATCCTTGCGGAAGTCGATGT | [3] | 104.14 |
| *Arg-2* | 144 | CGTGCTTTGGACAGACGAGA | AGAGCTCCTTAACGCCTAGC | [NM_001199704.1](https://www.ncbi.nlm.nih.gov/nuccore/NM_001199704.1) | 97.26 |
| *GAPDH* | 165 | CGTCCTCTCTGGCAAAGTCC | AAGATAGTGATGGCGTGCCC | [NM_204305.1](https://www.ncbi.nlm.nih.gov/nuccore/NM_204305.1) | 98.11 |
| *AID* | 87 | TGCTACCGCATCACATGGTT | GTTTGGGTAGGCACGAAGGA | [NM_001243222.1](https://www.ncbi.nlm.nih.gov/nuccore/NM_001243222.1) | 95.84 |
| *BAFF* | 291 | GGCAAGGTCTCCACTAGAGC | AGCCAAGGGACAATGCTTGA | [NM_204327.2](https://www.ncbi.nlm.nih.gov/nuccore/NM_204327.2) | 96.96 |
| *MyD88* | 131 | GGAGGATGGTGGTCGTCATT | CATGGTCTTGCACTTGACCG | [NM_001030962.4](https://www.ncbi.nlm.nih.gov/nuccore/NM_001030962.4) | 98.28 |

References

1. Khan S, Roberts J, Wu SB. Reference gene selection for gene expression study in shell gland and spleen of laying hens challenged with infectious bronchitis virus. Sci Rep. 2017;7(1):14271.

2. De Boever S, Vangestel C, De Backer P, Croubels S, Sys SU. Identification and validation of housekeeping genes as internal control for gene expression in an intravenous LPS inflammation model in chickens. Vet Immunol Immunopathol. 2008;122(3-4):312-7.

3. Lammers A, Wieland WH, Kruijt L, Jansma A, Straetemans T, Schots A, et al. Successive immunoglobulin and cytokine expression in the small intestine of juvenile chicken. Dev Comp Immunol. 2010;34(12):1254-62.

4. Paraskeuas V, Fegeros K, Palamidi I, Hunger C, Mountzouris KC. Growth performance, nutrient digestibility, antioxidant capacity, blood biochemical biomarkers and cytokines expression in broiler chickens fed different phytogenic levels. Anim Nutr. 2017;3(2):114-20.

5. Liu SQ, Wang LY, Liu GH, Tang DZ, Fan XX, Zhao JP, et al. Leucine alters immunoglobulin a secretion and inflammatory cytokine expression induced by lipopolysaccharide via the nuclear factor-kappaB pathway in intestine of chicken embryos. Animal. 2018;12(9):1903-11.
