# Supplementary material for: Bacteroides-derived isovaleric acid enhances mucosal immunity by facilitating intestinal IgA response in broilers
Source: J Anim Sci Biotechnol. 2023 Jan 6;14:4. doi: 10.1186/s40104-022-00807-y (PMC9817248; doi:10.1186/s40104-022-00807-y)
Supplement: Supplementary file 4 — Additional file 4: Table S4. IgA sequencing primers in this study [file 40104_2022_807_MOESM4_ESM.docx]

**Table S4** IgA sequencing primers in this study

| **Primer** | **Sequence** | |  |  |
| --- | --- | --- | --- | --- |
| Forward primer 1 | | CCGTGACGTTGGACGAG | |  |
| Forward primer 2 | | ATGGCGGCCGTGACGTTG | |  |
| Forward primer 3 | | GCGGCCGTGACGTTGGAC | |  |
| Reverse primer 1 | | TTTGGAGGTGAATATGGGGC | |  |
| Reverse primer 2 | | ACCGATGGTCTCCTTCACATC | |  |
| Reverse primer 3 | | TGGGGCAGACGCCATCCCCACC | |  |
